# Cerium(III) bromide hybrid with near-unity photoluminescence quantum efficiency for high-resolution and fast x-ray imaging

**DOI:** 10.1016/j.fmre.2025.02.007

**Published:** 2025-04-29

**Authors:** Jiance Jin, Kai Han, Yuzhen Wang, Maxim S. Molokeev, Liang Li, Zhiguo Xia

**Affiliations:** a State Key Laboratory of Luminescent Materials and Devices, Guangdong Provincial Key Laboratory of Fiber Laser Materials and Applied Techniques, Guangdong Engineering Technology Research and Development Center of Special Optical Fiber Materials and Devices, School of Materials Science and Engineering, South China University of Technology, Guangzhou 510641, China; b School of Physics and Optoelectronics, South China University of Technology, Guangzhou 510641, China; c Laboratory of Crystal Physics Kirensky, Institute of Physics Federal Research Center, KSC SB RAS, Krasnoyarsk 660036, Russia; d Department of Engineering Physics and Radioelectronic, Siberian Federal University, Krasnoyarsk 660041, Russia

**Keywords:** Scintillator, Cerium halide hybrids, Fast X-ray imaging, Short lifetime, Rare earth, Luminescent materials

## Abstract

Trivalent lanthanide Ce(III) with a typical allowed 4f – 5d transition generally exhibits short decay time in a nanosecond level, beneficial to fast X-ray imaging applications. Herein, we rationally design and synthesize two zero-dimensional Ce^3+^ based halide hybrids, namely [Emim]_3_CeBr_6_ and [Emmim]_3_CeBr_6_ (Emim = 1-ethyl-3-methylimidazolium; Emmim = 1-ethyl-2,3-dimethylimidazolium), both exhibiting intense broad-band violet-to-blue emission originated from Ce^3+^ with near-unity photoluminescence quantum yield. The short nanosecond decay time and the heavy atom effect further inspire us to explore their X-ray scintillation performance. Up to 48,000 photons MeV^-1^ light yield is derived together with the low detection limit (around 626 nGy s^-1^), and thus a composite film is fabricated to achieve dynamic fast X-ray imaging. This study enlarges the family of scintillators in Ce(III)-based halide hybrid with short decay time.

## Graphical abstract

Two cerium bromide hybrids [Emim]_3_CeBr_6_ and [Emmim]_3_CeBr_6_ (Emim = 1-ethyl-3-methylimidazolium; Emmim = 1-ethyl-2,3-dimethylimidazolium) exhibit intense 4f – 5d violet-to-blue emission from Ce^3+^ with photoluminescence quantum yield of 100%. The short nanosecond decay time and the heavy atom endows two compounds with high-resolution and fast X-ray imaging property with light yield up to 48,000 pH MeV^-1^ and low detection limit of 626 nGy s^-1^.

**Figure:**
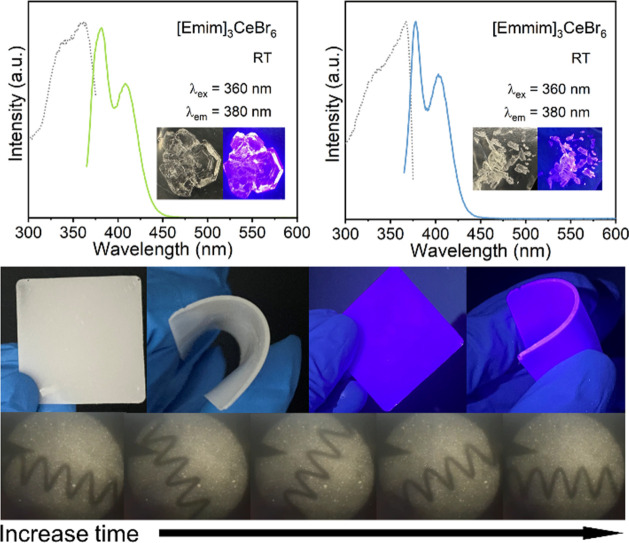

## 1 Introduction

X-ray scintillators, capable of absorbing high-energy X-ray radiation and converting to visible light signal, has raised great interest for their intense applications in medical imaging and industrial detection [[1], [2], [3]]. Conventional commercial X-ray scintillators are confined to oxide compounds, such as Bi_4_Ge_3_O_12_ (BGO), Y_3_Al_5_O_12_:Ce (YAG:Ce) and Lu_3_Al_5_O_12_:Ce (LuAG:Ce); however, high-temperature solid synthetic process will lead to much energy loss and require complex production processes and capital-intensive equipment [4]. Therefore, intensive attention has been paid to explore appropriate candidates obtained from convenient and simple process. Metal halides, especially emerging metal halide hybrids (MHHs), are noted for their convenient liquid phase synthesis become extremely appropriate [[5], [6], [7], [8]]. In fact, massive MHHs were reported to possess excellent X-ray scintillation property [[9], [10], [11]]. The detailed MHHs X-ray scintillators can be classified by the B-site metal ions. For transition B-site element, Mn^2+^ shows efficient X-ray scintillation property when coordinated to halogens to form an MHH; for instance, (C_38_H_34_P_2_)MnBr_4_ shows high light yield (LY) of around 80,000 photons MeV^-1^, and TPP_2_MnBr_4_ (TPP = tetraphenylphosphonium) ceramic possesses LY of 78,000 photons MeV^-1^ [12,13]. The other transition metal ion (Cu^+^) based MHHs also exhibit good X-ray scintillation property, such as (C_8_H_20_N)_2_Cu_2_Br_4_ with high LY of 91,300 photons MeV^-1^ [14]. For main group metal ions with ns^2^ electron configuration, Bmpip_2_SnBr_4_ and Bmpip_2_PbBr_4_ were also studied to possess intriguing X-ray excited radioluminescence (Bmpip = 1‑butyl‑1-methylpiperidinium) [15].

Nevertheless, fast scintillators receive intense attention because it is beneficial to good timing resolution, which reflects on the short decay time [16]. The ghost image during X-ray imaging can be greatly avoided utilizing scintillators with short decay time. Notably, Mn^2+^ based scintillators possess long emission lifetime up to millisecond although they exhibit appreciable LYs [17,18]. While many Cu^+^ based and main group ions based halides with self-trapped emission could have microsecond lifetime emission, a shorter decay is still imperative [[19], [20], [21]]. Recent studies show that lanthanide ion of Eu^2+^ possess both good LY and short lifetime [[22], [23], [24]]; however, it is still intrinsicly confined to the level of microsecond. Virtually, Ce^3+^ based compounds are known as the efficient scintillator with short lifetime [25]. The history can be tracked from star commercial oxide scintillator of LuAG:Ce to other Ce^3+^ doped halides, such as LaCl_3_:Ce^3+^ (25 ns), RbGd_2_Br_7_:Ce^3+^ (43 ns), and GdBr_3_:Ce^3+^ (20 ns) [[26], [27], [28]]. These materials were obtained by Bridgman technique, whose synthetic process involved high-temperature and a lot of time. Comparatively, Ce^3+^ based MHHs that are synthesized through low-temperature solution method are seldom investigated [29]. MHHs contains both organic and inorganic part, possessing the optical property from inorganic part and organically processable attribute from organic moiety [30].

Herein, we designed and prepared two cerium bromide hybrids by utilizing solution-volatilization process, namely C_18_H_33_N_6_CeBr_6_ ([Emim]_3_CeBr_6_) and C_21_H_39_N_6_CeBr_6_ ([Emmim]_3_CeBr_6_) (Emim = 1-ethyl-3-methylimidazolium; Emmim = 1-ethyl-2,3-dimethylimidazolium). Both compounds possess zero-dimensional (0D) structure with the isolated [CeBr_6_]^3-^ octahedron and organic cation. Upon 365 nm excitation, two materials emit 380 nm light with photoluminescence quantum yield (PLQY) up to 100%. The violet-to-blue emission is attributed to 4f - 5d of Ce^3+^ as confirmed by density functional theory (DFT) calculation. Interestingly, they also show very short decay time at nanosecond level, conducive to the scintillation application. Therefore, X-ray scintillation tests were conducted, demonstrating that [Emim]_3_CeBr_6_ and [Emmim]_3_CeBr_6_ possess high LYs with the value of 42,000 photons MeV^-1^ for [Emim]_3_CeBr_6_ and 48,000 photons MeV^-1^ for [Emmim]_3_CeBr_6_. The detection limit was 971 nGy s^-1^ for [Emim]_3_CeBr_6_, and 626 nGy s^-1^ for [Emmim]_3_CeBr_6_. Finally, dynamic fast X-ray imaging was successfully realized by the thin film fabricated via the mixure of [Emmim]_3_CeBr_6_ and photosensitive resin, showing negligible ghost imaging. This work enlarges the family of Ce^3+^ based MHHs, and sheds light on the efficient X-ray scintillator with a short decay time.

## 2 Material and methods/experiment

### 2.1 Materials and synthesis

The utilized reagents in the study were 1-ethyl-3-methylimidazolium bromide ([Emim]Br, 98% leyan), 1-ethyl-2,3-dimethylimidazolium bromide ([Emmim]Br, 98%, aladdin), CeBr_3_ (99%, aladdin), photosensitive resin (QIE FENG), anhydrous ethanol (EtOH, 99.5%, Macklin). All the chemicals were purchased and utilized without further purification.

[Emim]_3_CeBr_6_ and [Emmim]_3_CeBr_6_ were synthesized by solution-volatilization method. For [Emim]_3_CeBr_6_, [Emim]Br (3 mmol), and CeBr_3_ (1 mmol) were added into a 20 mL glass bottle. Then 4 mL EtOH were added into the glass bottle. The glass bottle was heated to 70 °C under continuous stirring until homogeneous colorless solution was reached. After that, the bottle containing solution was placed at room-temperature (RT) for crystal growth. Colorless transparent crystals were formed within three days. For [Emim]_3_CeBr_6_, the synthetic method is the same except for the change of organic reagent to [Emmim]Br (3 mmol).

Note that all the synthetic process was conducted in the glove box filled with N_2_ atmosphere.

Fabrication of flexible film. The single crystals of [Emmim]_3_CeBr_6_ were thoroughly grounded in a mortar to yield homogeneous powder, respectively. 1 g [Emmim]_3_CeBr_6_ powder was mixed with the photosensitive resin to form a turbid liquid. The liquid was then carefully placed onto a clean glass or a white substrate. The glass and the white substrate was placed at RT for 10 min under UV light irradiating to eventually bring about the thin film. The film fabrication was also conducted in the glove box filled with N_2_ atmosphere.

### 2.2 Single-crystal X-ray diffraction

Single-crystal data of [Emim]_3_CeBr_6_ and [Emmim]_3_CeBr_6_ was collected on a XtaLAB P200 FR-X-ray single-crystal diffractometers equipped with the Pilatus 200k detector, and was tested by Mo Kα radiation (*λ* = 0.71073 Å) at 100 K for [Emim]_3_CeBr_6_ and 110 K for [Emmim]_3_CeBr_6_. The structures were solved by direct methods and refined by full-matrix least-squares on *F*^2^ using the *SHELX*-2018 program package [31]. All non-hydrogen atoms were refined anisotropically, and the hydrogen atoms connected to C atoms were located at geometrically calculated positions. The crystallographic data and details of structural refinements are listed in Table S1. Detailed bond lengths and angles were listed in Tables S2,3. Data concerning H-bond interactions were shown in Tables S4,5.

### 2.3 Characterization

The powder X-ray diffraction (PXRD) data of [Emim]_3_CeBr_6_ and [Emmim]_3_CeBr_6_ for Rietveld analysis were collected at room temperature with a Bruker D8 ADVANCE powder diffractometer (Cu-Kα radiation) and linear VANTEC detector. The step size of 2θ was 0.011°, and the counting time was 2 s per step. All peaks were indexed by monoclinic cell (*P*2_1_/*c*) and triclinic (*P*-1), for [Emim]_3_CeBr_6_ and [Emmim]_3_CeBr_6_ respectively. Therefore these structures were taken as starting model for Rietveld refinement which was performed using TOPAS 4.2 [32]. All atom coordinates and thermal parameters were fixed during refinement due to low ratio of observed strong peaks to number of refined parameters. Final refinements which account cell parameters and profile parameters were stable and gave low R-factors.

Solid-state optical diffuse reflectance spectra were recorded on a UV–Vis-NIR spectrometer at RT in the range of 800 - 250 nm (Hitachi High-Tech Science Corporation, UH4150). A BaSO_4_ plate with 100% reflectance was utilized as a standard. The absorption data were calculated from the reflectance spectra by using the Kubelka-Munk function *α*/*S* = (1 - *R*)^2^/2*R* [33], where *α* is the absorption coefficient, *S* is the scattering coefficient, and *R* refers to the reflectance. X-ray photoelectron spectroscopy (XPS) was performed on Thermo Scientific K-Alpha. Energy Dispersive Spectrometer (EDS) and elemental distribution mapping were obtained by a JSM-7900F scanning electron microscope (SEM) equipped with a Bruker XFlash 6–100 EDS detector. Elemental analysis was tested on Elementar UNICUBE. Thermogravimetric (TG) analyses were performed on a NETZSCH TG 209F3 with a heating rate of 10 K min^-1^ under N^2^ atmosphere. Photoluminescence (PL), PL excitation (PLE) and PL decay spectra were performed employing a FLS1000 fluorescence spectrophotometer (Edinburgh Instruments Ltd., U. K.). Excitation spectra for both compounds were obtained when the emission wavelength was monitored at around 380 nm and 405 nm. Photoluminescence quantum yields (PLQYs) were tested via an absolute PL quantum yield spectrometer (Quantaurus-QY Plus C13534–11, Hamamatsu Photonics).

### 2.4 Hirshfeld surface analyses

The intermolecular interactions for [Emim]_3_CeBr_6_ and [Emmim]_3_CeBr_6_ were studied through Hirshfeld surface analysis by utilizing Crystal Explore 17 [[34], [35], [36], [37], [38]]. The Hirshfeld surface of a crystal molecule is fabricated by partitioning space in the crystal into regions where the ratio of electron density of a sum of spherical atoms for molecule (the promolecule) is equal to 0.5. *d_e_* is the distance from the Hirshfeld surface to the nearest nucleus outside the surface, while *d_i_* refers to the distance from Hirshfeld surface to internal nearest nucleus. *d_norm_* is the sum of *d_e_* and *d_i_* which are both normalized by van der Waals radii (*r^vdw^*). The highlighted red color on the Hirshfeld surface *d_norm_* indicates that the intermolecular contacts are closer than the sum of their van der Waals radii. The white highlights denote the interactions around the sum of *r^vdw^*, while the blue highlights represent the longer contacts. The 2D fingerprint plots are used to summarize the intermolecular interactions by plotting the distribution of points derived from the Hirshfeld surface [39]. On the 2D fingerprint plots, each point corresponds to a unique (*d_e_, d_i_*) pair, and their color corresponds to the contribution of the weak interactions. Blue color refers to a small contribution to the surface, while red color indicates the greatest contribution.

### 2.5 X-ray scintillation performance

Radioluminescence spectra and detection limit at RT were recorded by FLS1000 fluorescence spectrophotometer (Edinburgh Instruments Ltd., UK). All compounds were closely attached to the circular window of an integrating sphere with an X-ray source (Amptek Mini-X tube with a Mo target and 6 W maximum power output). Light yield (LY) value can be calculated via the ratio of the total emitted photon number of [Emim]_3_CeBr_6_ and [Emmim]_3_CeBr_6_ and reference sample (LuAG:Ce, 25,000 photons MeV^-1^) at equivalent absorbed X-ray energy. X-ray imaging was acquired by using a CMOS camera (KnightCam S455) with 9568 ×  6380 pixels and a 3.76-μm pixel size.

### 2.6 Computational methodology

Density functional theory (DFT) calculations were performed for [Emim]_3_CeBr_6_ and [Emmim]_3_CeBr_6_ samples. Projector augmented wave (PAW) method based on a generalized gradient approximation (GGA) was adopted in the calculation, and the Perdew-Burke-Ernzerhof (PBE) format was utilized for the exchange correlation potential [40,41]. The supercells based on the primitive cell were utilized in the calculation with the cutoff energy of a plane-wave basis set as 520 eV MonkhorstPack mesh of k-point was set as 2 × 2 × 2 for [Emim]_3_CeBr_6_ and 3 × 3 × 2 for [Emmim]_3_CeBr_6_, respectively. The atoms in each compound were fully relaxed until the Hellmann-Feynman forces on them were within 0.01 eV/Å. The electronic iteration convergence was set as 10^–5^ eV Before the calculation of bandgap and density of state (DOS), structural optimization was firstly performed using the PBE exchange correlation potential. As for the calculation of DOS, the k-point was set as 4 × 4 × 4 for each sample. To consider the strong Coulomb repulsion for the Ce-4*f* electrons, Hubbard correction was adopted to PBE (GGA+*U*) [42].

## 3 Results and discussion

### 3.1 Crystal structure and phase analysis

**Fig. 1:**
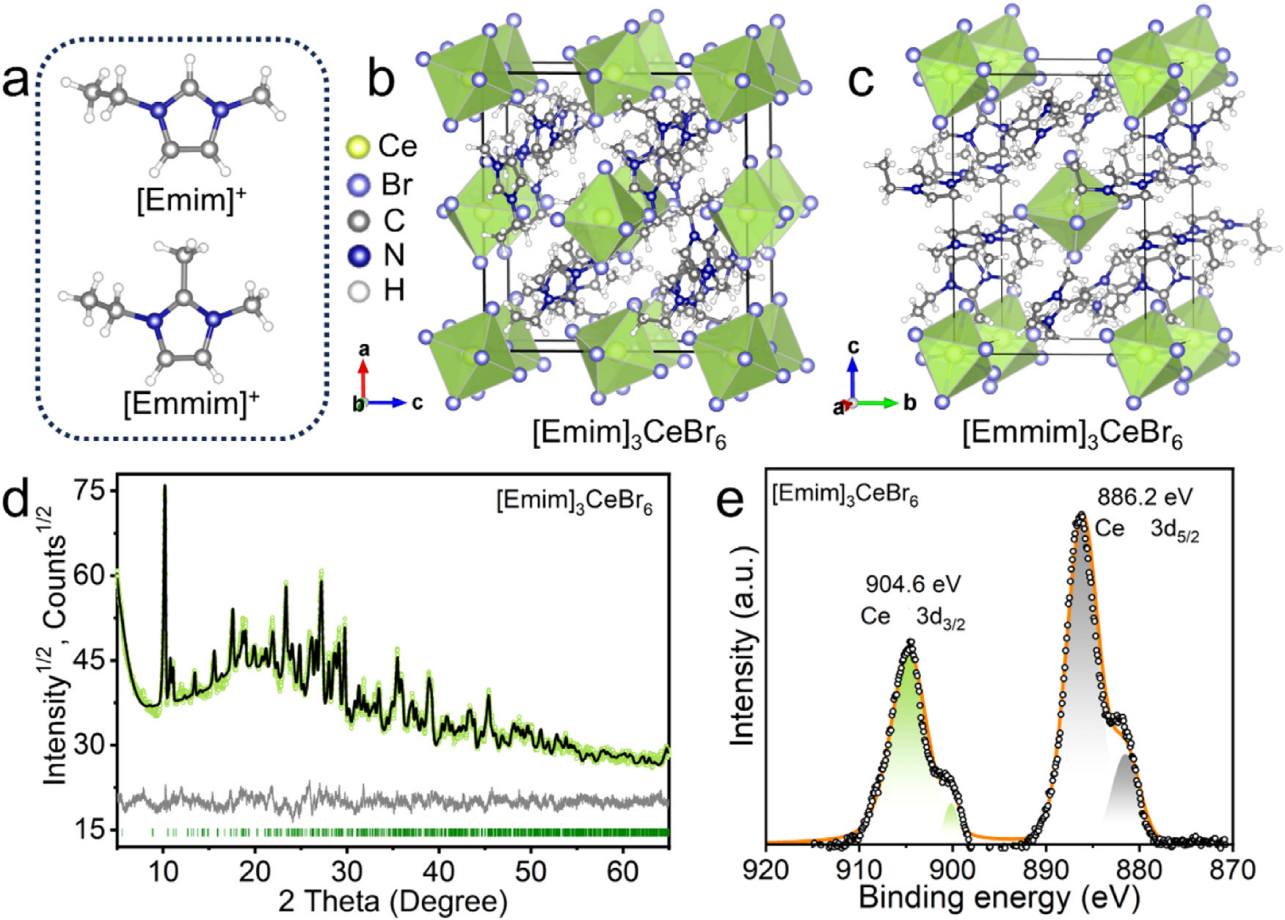
(a) Structure of Emim and Emmim cation used in the synthesis. Crystal structure of [Emim]_3_CeBr_6_ (b) and [Emmim]_3_CeBr_6_ (c). (d) X-ray refinement with difference Rietveld plot of [Emim]_3_CeBr_6_. (e) High-resolution X-ray photoelectron spectroscopy (XPS) spectrum of Ce 3d for [Emim]_3_CeBr_6_.

Both [Emim]_3_CeBr_6_ and [Emmim]_3_CeBr_6_ have been synthesized via a solution-volatilization process in a glove box filled with N_2_ atmosphere. They exhibit 0D structure with the same isolated [CeBr_6_]^3-^ anion charge-compensated by different imidazolium cations as shown in Fig. 1a [43]. [Emim]_3_CeBr_6_ crystallizes in the monoclinic space group of *P*2_1_/*c* with *a* = 15.8919(5) Å, *b* = 12.7719(4) Å, *c* = 15.1052(5) Å, *β* = 90.458(3)°. (Fig. 1b, and Table S1). For [Emmim]_3_CeBr_6_, it belongs to the *P*-1 triclinic space group with *a* = 10.04200(10) Å, *b* = 10.8313(2) Å, *c* = 15.8543(2) Å, *α* = 90.7380(10)°, *β* = 92.1860(10)°, *γ* = 106.9450(10)°. (Fig. 1c, and Table S1). The Ce-Br bond lengths vary from 2.8971(6) to 2.9246(5) Å in [Emim]_3_CeBr_6_ (Table S2) and 2.9049(4) to 2.9439(4) Å in [Emmim]_3_CeBr_6_ (Table S3), which are comparable with other reported cerium bromide hybrids [44]. Additionally, abundant H-bond interactions exist in both compounds leading to the construction of 3D supramolecular structures (Figures S1,2 and Tables S4, 5). The H-bond interactions can be also reflected from the 2D fingerprint plots derived from Hirshfeld surface analysis (Figures S3,4) [[34], [35], [36], [37], [38], [39]]. We performed Hirshfeld surface analysis by selecting [CeBr_6_]^3-^ anion to calculate the contribution of H-bond (Figures S3a,4a; inside atom: Br; outside atom: H). The red color indicates the great contribution while the blue one shows the small contribution. From the 2D fingerprint plots (Figures S3b, 4b), the contribution of H-bond interaction is 97.1% for [Emim]_3_CeBr_6_ and 95.7% for [Emmim]_3_CeBr_6_, indicating the rich H-bond interaction in both compounds. Elemental analysis shows that the experimental mass fraction of C, N, H is 22.562%, 8.824%, and 5.801% for [Emim]_3_CeBr_6_, respectively (theoretically, C: 22.685%; N: 8.818%; H: 3.490%); the mass fraction of C, N, H is 25.096%, 8.390%, and 6.212% for [Emmim]_3_CeBr_6_, respectively (theoretically, C: 25.346%; N: 8.445%; H: 3.950%). Notably, the increase in H content is due to the absorption of water (H_2_O) during the characterization process. The experimental results of C and N match well with those of theoretical one, indicating the exact content of the materials.

To confirm the purity of both compounds, powder X-ray diffraction (PXRD) refinement was performed (Figs. 1d and S5). The low fit parameters of *R*_p_, *R*_wp_, *R*_B_ and χ^2^ suggest the purity of two compounds and the reliability of the refinements (Table S6). The refined single-crystal parameters were also listed, compable to the results from single-crystal X-ray diffraction (Table S1). X-ray photoelectron spectroscopy (XPS) results for two compounds were shown in Figs. 1e and S6. For [Emim]_3_CeBr_6_, the binding energy at 904.6 eV and 886.2 eV is ascribed to 3d_3/2_ and 3d_5/2_ of Ce, respectively. For [Emmim]_3_CeBr_6_, the binding energy at 904.2 eV and 885.9 eV is ascribed to 3d_3/2_ and 3d_5/2_ of Ce, respectively. We also performed scanning electron microscope (SEM) and energy-dispersive spectroscopy (EDS) elemental mapping, which shows that the Ce, N, and Br elements are uniformly distributed (Figures S7,8). These results indicate the exisence of Ce^3+^ and the successful syntheses of two title compounds. Furthermore, TG curve was tested for two compounds showing that both possess good thermal stability until the temperature reached 300 °C (Figure S9). Notably, the mass loss at around 100 °C is attributed to the escape of the absorped water owing to the hygroscopicity of Ce^3+^ based halides.

### 3.2 Photoluminescence properties

**Fig. 2:**
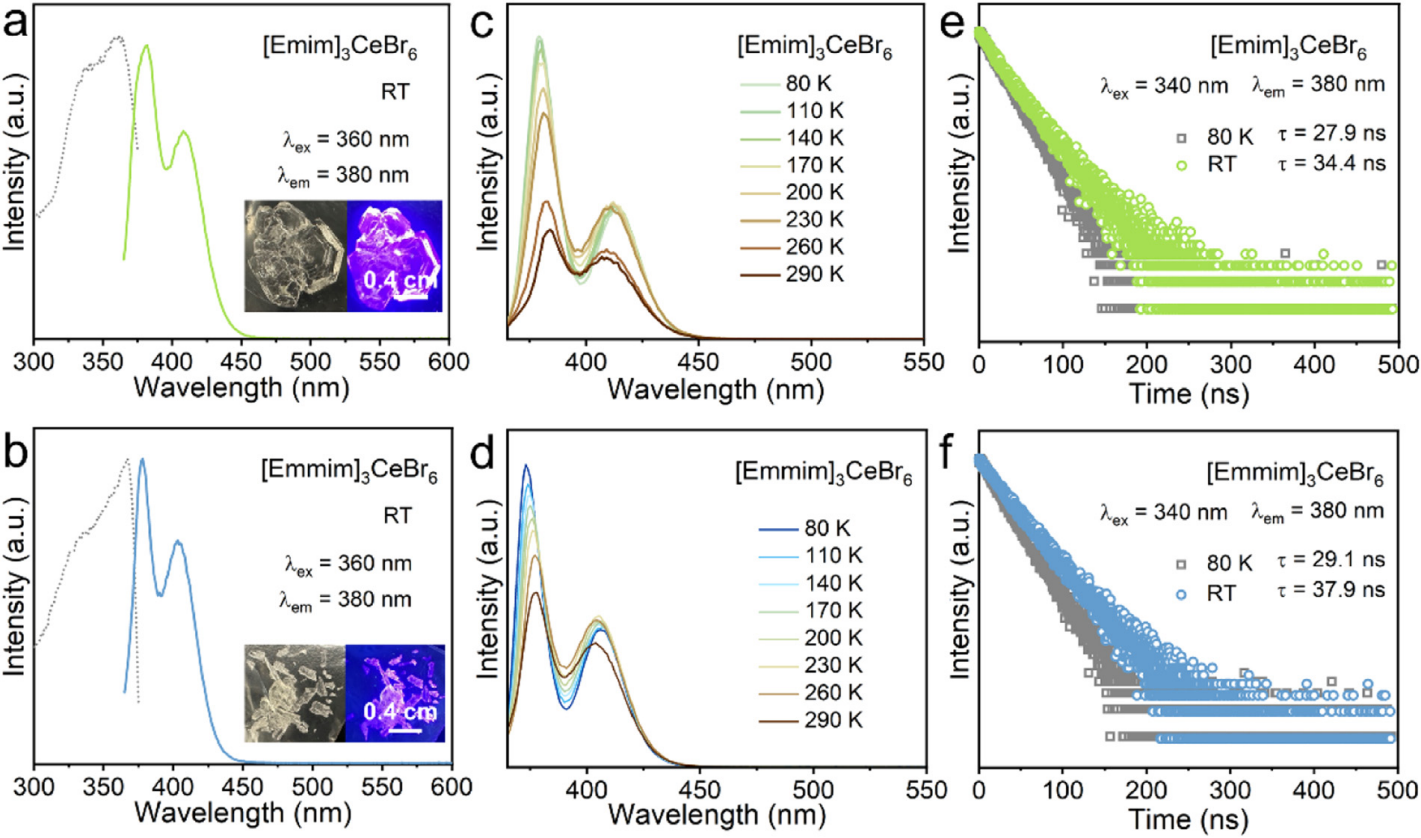
PLE and PL spectra of [Emim]_3_CeBr_6_ (a) and [Emmim]_3_CeBr_6_ (b); inset shows the single-crystal under natural light and 365 nm UV light. Temperature-dependent PL spectra of [Emim]_3_CeBr_6_ (c) and [Emmim]_3_CeBr_6_ (d) under 360 nm excitation. PL decay spectra at 80 K and RT upon 340 nm laser excitation and monitored at 380 nm emission for [Emim]_3_CeBr_6_ (e) and [Emmim]_3_CeBr_6_ (f).

Photoluminescence excitation (PLE) and PL spectra for [Emim]_3_CeBr_6_ and [Emmim]_3_CeBr_6_ were shown in Figs. 2a and b. Two compounds emitted violet-to-blue light with the maximum emission wavelength of 380 nm under the excitation of 360 nm at room-temperature (RT). They exhibit characteristic dual emission peaks at around 380 and 408 nm, which is comparable with other Ce^3+^ based halides [29,44]. The two emission peaks are attributed to the transition between 5d excited states and the spin-orbit split of 4f ground state (namely, 2F_5/2_ and 2F_7/2_ levels) of Ce^3+^ [45]. The excitation spectra for both compounds when the emission wavelength was set at around 400 nm were shown in Figures S10,11, which is in accordance with the results monitored at 380 nm emission (Figs. 2a and b). Photoluminescence quantum yield (PLQY) for both compounds was measured to be near unity.

To get deep insights into the luminescent mechanism, temperature-dependent PL spectra were firstly measured from 80 K to RT. The PL intensity shows remarkable enhancement under low-temperature while no peak shifts occur for two compounds, indicating only one emission mechanism, i.e. 4f - 5d transition of Ce^3+^ [44]. Additionally, exciton binding energy (*E*_b_) could reveal the effect brought by the highly localized exciton, which can be estimated from the Arrhenius formula [46]:

(1) $$I\left(T\right)=\frac{I_{0}}{1+Aexp(-E_{b}/2k_{B}T)}$$

where $I_{0}$ is the intensity at 80 K, $E_{b}$ is the exciton binding energy, $k_{B}$ is the Boltzmann constant, and *T* is the temperature. *E*_b_ is calculated to be 142.56 meV for [Emim]_3_CeBr_6_ and 60.2 meV for [Emmim]_3_CeBr_6_ (Figure S12). The high *E*_b_ indicates the localized characteristic of Ce-4f orbital and the 0D crystal structure of [CeBr_6_]^3-^.

PL lifetime monitored at the emission of 380 nm for both compounds at 80 K and RT was also tested (Figs. 2e and f). Under low-temperature, [Emim]_3_CeBr_6_ possesses short decay time of 27.9 ns; and [Emmim]_3_CeBr_6_ also shows short decay time of 29.1 ns. However, when the temperature increased to RT, the lifetime underwent abnormal change, showing slight lengthening with the value of 34.4 ns for [Emim]_3_CeBr_6_ and 37.9 ns for [Emmim]_3_CeBr_6_. The PL lifetime of 408 nm emission was also tested. The lifetime is 28.2 ns at 80 K and 37.5 ns at RT for [Emim]_3_CeBr_6_ (Figure S13). As for [Emmim]_3_CeBr_6_, the lifetime is 29.3 ns at 80 K and 38.6 ns at RT (Figure S14). Negligible change exists for two compounds when monitored at different emission wavelengths. Notably, All the lifetime presents a mono-exponential fit both at low-temperature and RT, which further indicates the classic 4f - 5d emission from Ce^3+^ located at the single crystallographic site.

### 3.3 Absorption measurement and DFT calculation

**Fig. 3:**
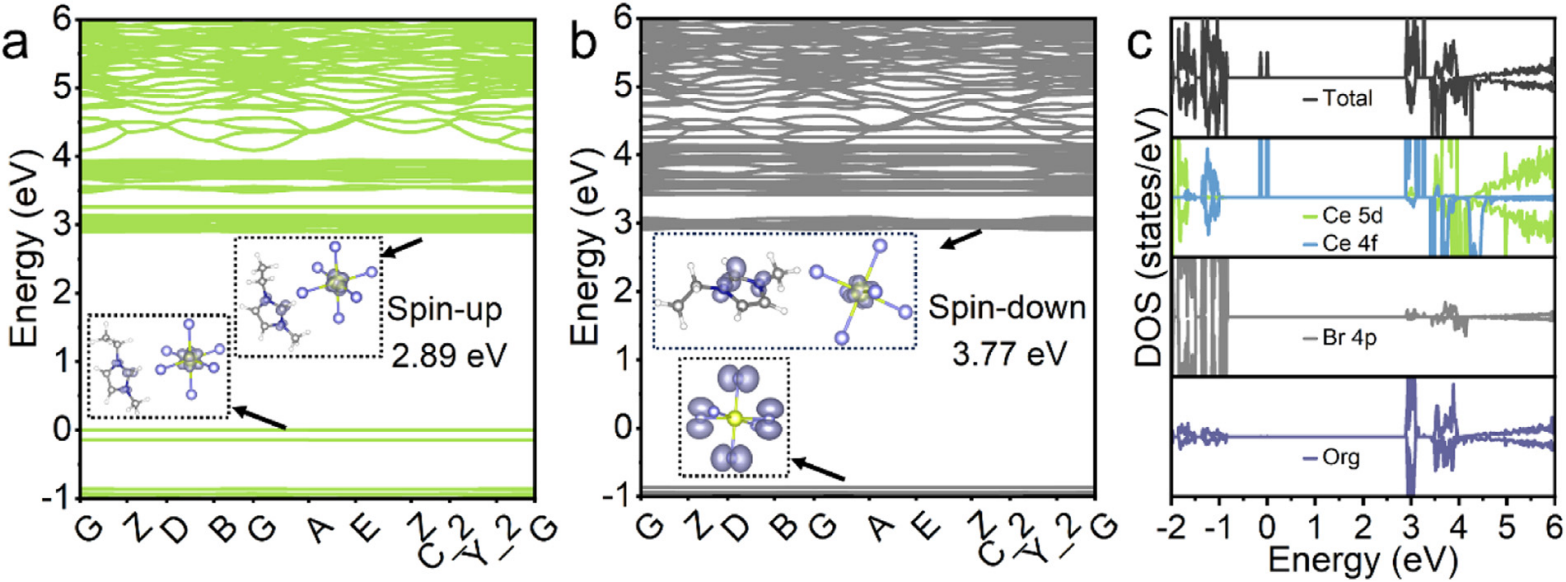
Calculated spin-up (a) and spin-down (b) band structure of [Emim]_3_CeBr_6_ with the bandgap value of 2.89 and 3.77 eV, respectively; inset shows the charge-density at VBM and CBM. (c) Density of state (DOS) of [Emim]_3_CeBr_6_.

The experimental bandgap value was obtained from the absorption spectra for two compounds (Figures S15,16), and the values are 3.63 eV for [Emim]_3_CeBr_6_ and 3.93 eV for [Emmim]_3_CeBr_6_, respectively. Furthermore, we performed density functional theory (DFT) calculation to address the luminescent mechanism from 4f - 5d of Ce^3+^. For [Emim]_3_CeBr_6_, the calculated bandgap is 2.89 eV for spin-up band structure (Fig. 3a), and 3.77 for spin-down band structure (Fig. 3b). With regard to [Emmim]_3_CeBr_6_, the spin-up bandgap is 2.92 eV (Figure S17a) and the spin-down bandgap is 3.77 eV (Figure S17b). The charge density of valence band maximum (VBM) and conduction band minimum (CBM) for both compounds demonstrates that 4f and 5d orbitals of Ce^3+^ play significant roles in contributing the spin-up band structure at VBM and CBM. For spin-down band structure, the VBM for two compounds is occupied by the 4p of Br^-^ while the CBM is determined by cation and 4f of Ce^3+^. Density of state for both compounds were also shown in Figs. 3c and S17c. The VBM is occupied by 4f of Ce^3+^, while the CBM is composed of 5d and 4f of Ce^3+^. The 4f – 4f transition is parity-forbidden and causes a long lifetime emission [44]. Therefore, given that the nanosecond-level short lifetime of two compounds, it can be confirmed that the 4f – 5d transition results in the violet-to-blue emission. It also should be noted that organic cation of [Emim]^+^ and [Emmim]^+^ also contributes to the CBM, which is very common for the conjugate attribute [[47], [48], [49]].

### 3.4 Scintillation study

**Fig. 4:**
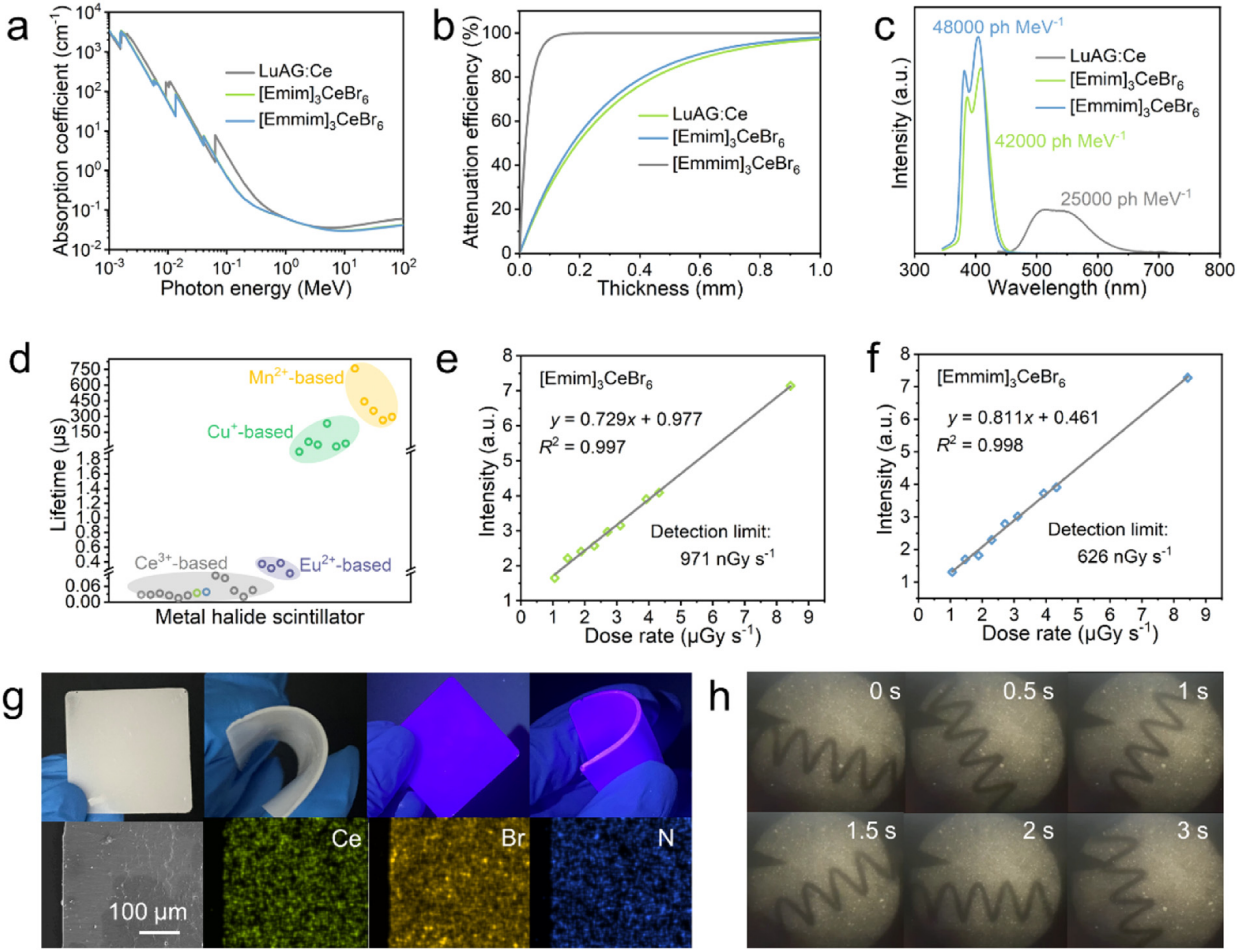
(a) Absorption coefficients of LuAG:Ce, [Emim]_3_CeBr_6_ and [Emmim]_3_CeBr_6_ as a function of photon energy from 1 keV to 100 MeV. (b) Attenuation efficiency of LuAG:Ce, [Emim]_3_CeBr_6_ and [Emmim]_3_CeBr_6_ as a function of sample thickness. (c) Radioluminescence spectra of LuAG:Ce, [Emim]_3_CeBr_6_ and [Emmim]_3_CeBr_6_. (d) Lifetimes of Mn^2+^, Cu^+^, Eu^2+^ and Ce^3+^-based metal halide scintillators. (e) Radioluminescence intensity as a function of X-ray dose rate for [Emim]_3_CeBr_6_. (f) Radioluminescence intensity as a function of X-ray dose rate for [Emmim]_3_CeBr_6_. (g) As-prepared thin film based on [Emmim]_3_CeBr_6_ under natural light and UV light and its SEM and EDS elemental mapping images of N, Br, Ce. (h) Dynamic X-ray images recording the rotating spring and the static blade. Note that the dose rate is 11.50 mGy s^-1^ for the LY test and X-ray imaging.

The short lifetime and heavy atom property for Ce^3+^ based compounds inspires us to design the new scintillators and study their scintillation property. In fact, all-inorganic Ce^3+^ doped halides were reported to possess excellent gamma-ray scintillation [25]; however, scintillators based on the Ce^3+^-contained MHHs were seldom discussed, let alone X-ray scintillator. The absorption coefficiency for two compounds and commercial scintillator of LuAG:Ce^3+^ was calculated and compared, which shows comparable absorption coefficiency between two title compounds and LuAG:Ce^3+^ (Fig. 4a). Moreover, X-ray attenuation efficiency was estimated, and a favorable thickness as the function of photon energy of 17.5 keV was chosen to evaluate the scintillation performance (Fig. 4b). [Emim]_3_CeBr_6_ and [Emmim]_3_CeBr_6_ exhibit similar X-ray luminescence, whose intensity is more intense than that of LuAG:Ce^3+^. The X-ray luminescent peaks for two compounds are also similar to that of PL peaks, indicating the same luminescent mechanism, that is 4f - 5d emission from Ce^3+^. The LY were calculated to be 42,000 photons MeV^-1^ for [Emim]_3_CeBr_6_ and 48,000 photons MeV^-1^ for [Emmim]_3_CeBr_6_, respectively (Fig. 4c). These values are much higher than that of commercial scintillator LuAG:Ce^3+^. The X-ray irradiation stability was tested, showing that [Emmim]_3_CeBr_6_ possesses better stability upon strong X-ray irradiation (Figure S18).

According to previous report, recent metal halide scintillators can be classified into transition metal ion-based, and main group metal ion-based materials. Specifically, the commonly studies of metal halide scintillators focus on Mn^2+^ and Cu^+^ for transition metal ions that have remarkable LYs (Table S7) [[12], [13], [14],[19], [20], [21],[50], [51], [52], [53]]. However, as shown in Fig. 4d the lifetimes of Mn^2+^ are very long and up to millisecond level, which may cause ghost image during X-ray imaging application. Additionally, even Cu^+^ possesses microsecond lifetime, it remains urgent to explore scintillators with shorter lifetimes. Therefore, rare-earth ion-based scintillators, especially for Eu^2+^ and Ce^3+^-doped or contained compounds, possess very short decay time, have become an appropriate candidate. Previous researches show that the lifetime of Eu^2+^ based MHHs could reach around 100 nanoseconds, but the LY for these compounds still require big improvement (Table S7 and Fig. 4d) [22,23]. Additionally, Ce^3+^ could be successfully doped into metal halide host to produce much shorter lifetime emission (Table S8 and Fig. 4d) [[26], [27], [28],[54], [55], [56], [57], [58], [59], [60]]. Notably, Therefore, [Emim]_3_CeBr_6_ and [Emmim]_3_CeBr_6_ exhibiting both short lifetimes and great LY values are promising X-ray scintillators.

The detection limit was also derived when the signal-to-noise ratio equals 3. The value is 971 nGy s^-1^ for [Emim]_3_CeBr_6_ and 626 nGy s^-1^ for [Emmim]_3_CeBr_6_ (Figs. 4e,f). These detection limit values are lower than the dose of 5.5 μGy s^−1^ for the medical treatment requirement of X-ray diagnostics [61,62]. Moreover, to realize fast X-ray scintillation, a thin film based on [Emmim]_3_CeBr_6_ was fabricated by mixing its powder with photosensitive resin. The as-prepared composite film exhibits flexible property, and violet-to-blue emission upon UV excitation, indicating that the original [Emmim]_3_CeBr_6_ was not destroyed (Fig. 4 g). Furthermore, such thin film also overcomes deliquescence for Ce^3+^ based halides, which maintains luminescence after a month in air. SEM and EDS elemental mapping was performed upon thin film of [Emmim]_3_CeBr_6_, indicating the good quality of film and uniform distribution of element. To examine the fast X-ray imaging, we designed a dynamic X-ray imaging device utilizing clock model with a rotating spring and static blade. As is shown in Video S1 and Fig. 4h, a clear image of spring and blade can be observed upon X-ray irradiation and no obvious ghost image exists during the rotation. Finally, we examined spatial resolution by utilizing a standard line-pair card (lp mm^-1^); A value of around 7 lp mm^-1^ was observed for [Emmim]_3_CeBr_6_ based thin film (Figure S19). These result shows that such materials with short decay time are promising candidate applied to high-resolution and fast X-ray imaging.

## 4 Conclusions

In conclusion, two Ce^3+^ based halide hybrids were synthesized, namely [Emim]_3_CeBr_6_ and [Emmim]_3_CeBr_6_. Both exhibit zero-dimensional crystal structure and emit violet-to-blue emission when excited by UV light. Their luminescent mechanism is attributed to 4f - 5d transtion from Ce^3+^. Intriguingly, the PLQYs were 100% for both compounds with very short decay of 34.4 ns for [Emim]_3_CeBr_6_ and 37.9 ns for [Emmim]_3_CeBr_6_, respectively. The heavy atom and the nanosecond level decay times of two compounds help to their X-ray scintillation property, and the LY was calculated to be 42,000 photons MeV^-1^ for [Emim]_3_CeBr_6_ and 48,000 photons MeV^-1^ for [Emmim]_3_CeBr_6_, respectively. The detection limit is 971 nGy s^-1^ for [Emim]_3_CeBr_6_ and 626 nGy s^-1^ for [Emmim]_3_CeBr_6_. Finally, a composite film based on [Emmim]_3_CeBr_6_ was fabricated and applied to fast X-ray imaging owing to the intrinsic short decay time. This study enlarges the family of hybrid rare earth halides scintillators with short decay time and expand the scintillation and imaging applications.

## CRediT authorship contribution statement

X.Z.G. and J.J.C. conceived the initial concept. J.J.C. prepared the sample and processed the experimental data. H.K. helped in the dynamic X-ray imaging test. M.M.S helped in the X-ray refinement results. L.L helped in crystal synthesis. J.J.C. wrote the paper. W.Y.Z helped in revising the paper. X.Z.G. supervised the work and revised the paper. All authors discussed and edited the paper.

## Declaration of competing interest

The authors declare that they have no conflicts of interest in this work.

## Appendix Supplementary materials

Image, application 1

Image, video 2 dummy alt text
