# Supplementary material for: Cerium(III) bromide hybrid with near-unity photoluminescence quantum efficiency for high-resolution and fast x-ray imaging
Source: Fundam Res. 2025 Apr 29;6(3):1794–800. doi: 10.1016/j.fmre.2025.02.007 (PMC13247475; doi:10.1016/j.fmre.2025.02.007)
Supplement: Supplementary file 1 [file mmc1.docx]

**Cerium(III) Bromide Hybrid with Near-Unity Photoluminescence Quantum Efficiency for High-Resolution and Fast X-ray Imaging**

Jiance Jin^a^, Kai Han^a,b^, Yuzhen Wang^a,b^, Maxim S. Molokeev^c,d^, Liang Li^a,b^, Zhiguo Xia ^a,b,*^

*^a^ State Key Laboratory of Luminescent Materials and Devices, Guangdong Provincial Key Laboratory of Fiber Laser Materials and Applied Techniques, School of Materials Science and Engineering, South China University of Technology, Guangzhou 510641, China*

*^b^* *School of Physics and Optoelectronics, South China University of Technology, Guangzhou 510641, China*

*^c^* *Laboratory of Crystal Physics Kirensky, Institute of Physics Federal Research Center, KSC SB RAS, Krasnoyarsk, 660036, Russia*

*^d^* *Department of Engineering Physics and Radioelectronic, Siberian Federal University, Krasnoyarsk, 660041, Russia*

*^*^ Corresponding author:* [*xiazg@scut.edu.cn*](mailto:xiazg@scut.edu.cn) *(Zhiguo Xia).*

## Figures


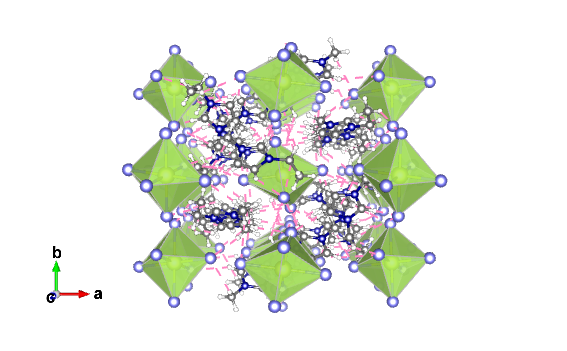


**Figure S1.** 3D supramolecular structure connected via H-bond for [Emim]_3_CeBr_6_.


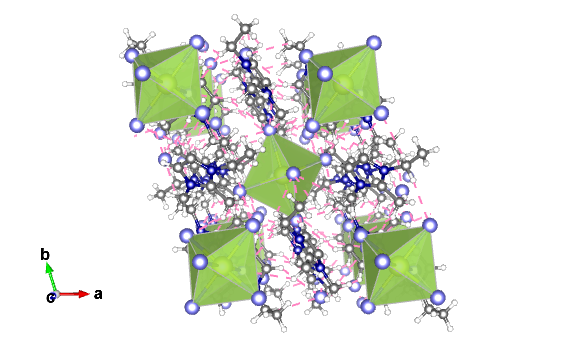


**Figure S2.** 3D supramolecular structure connected via H-bond for [Emmim]_3_CeBr_6_.


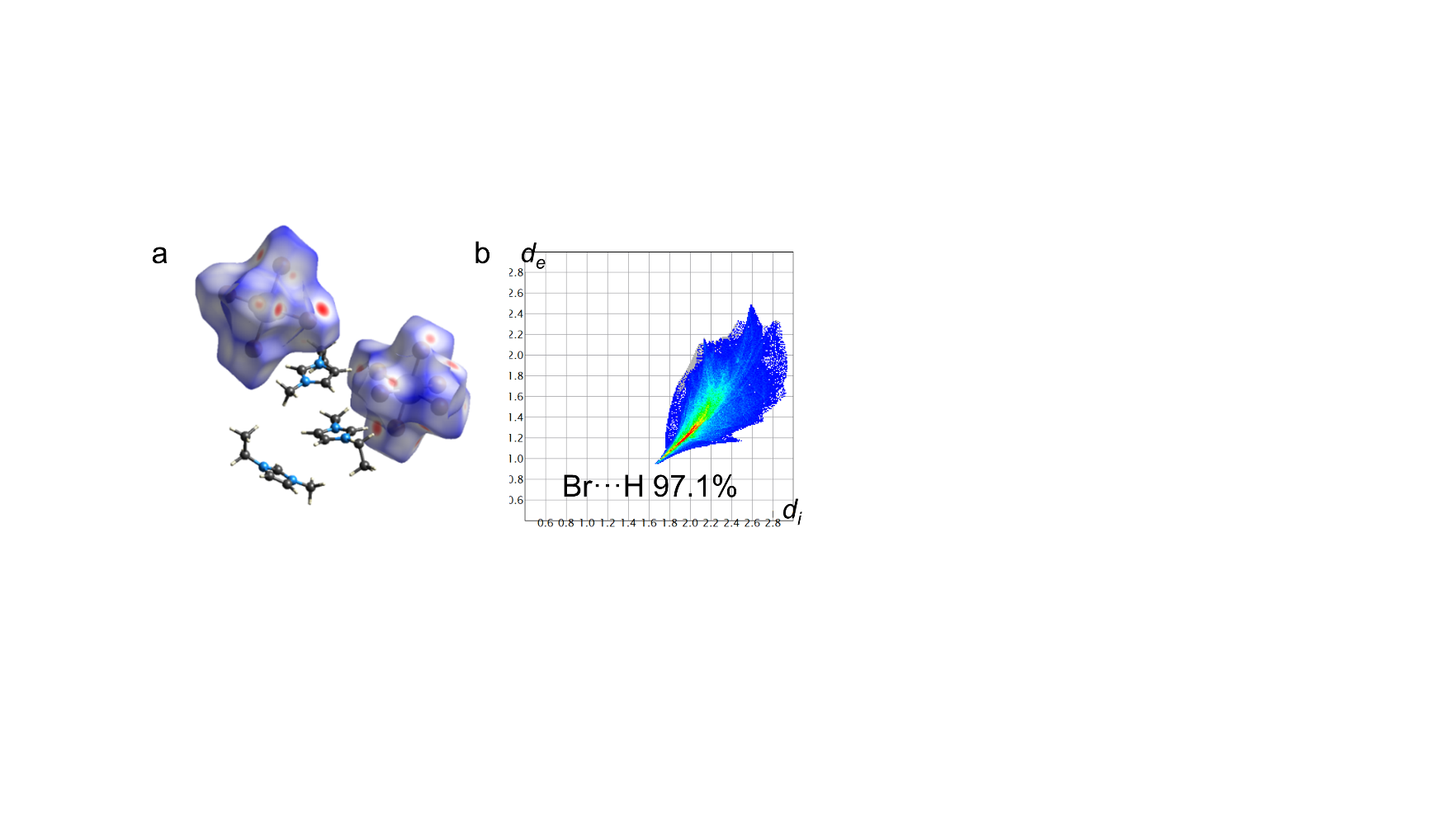


**Figure S3.** Hirshfeld surface analysis (a) and 2D fingerprint plot (b) of [Emim]_3_CeBr_6_ showing Br···H interaction (97.1%).


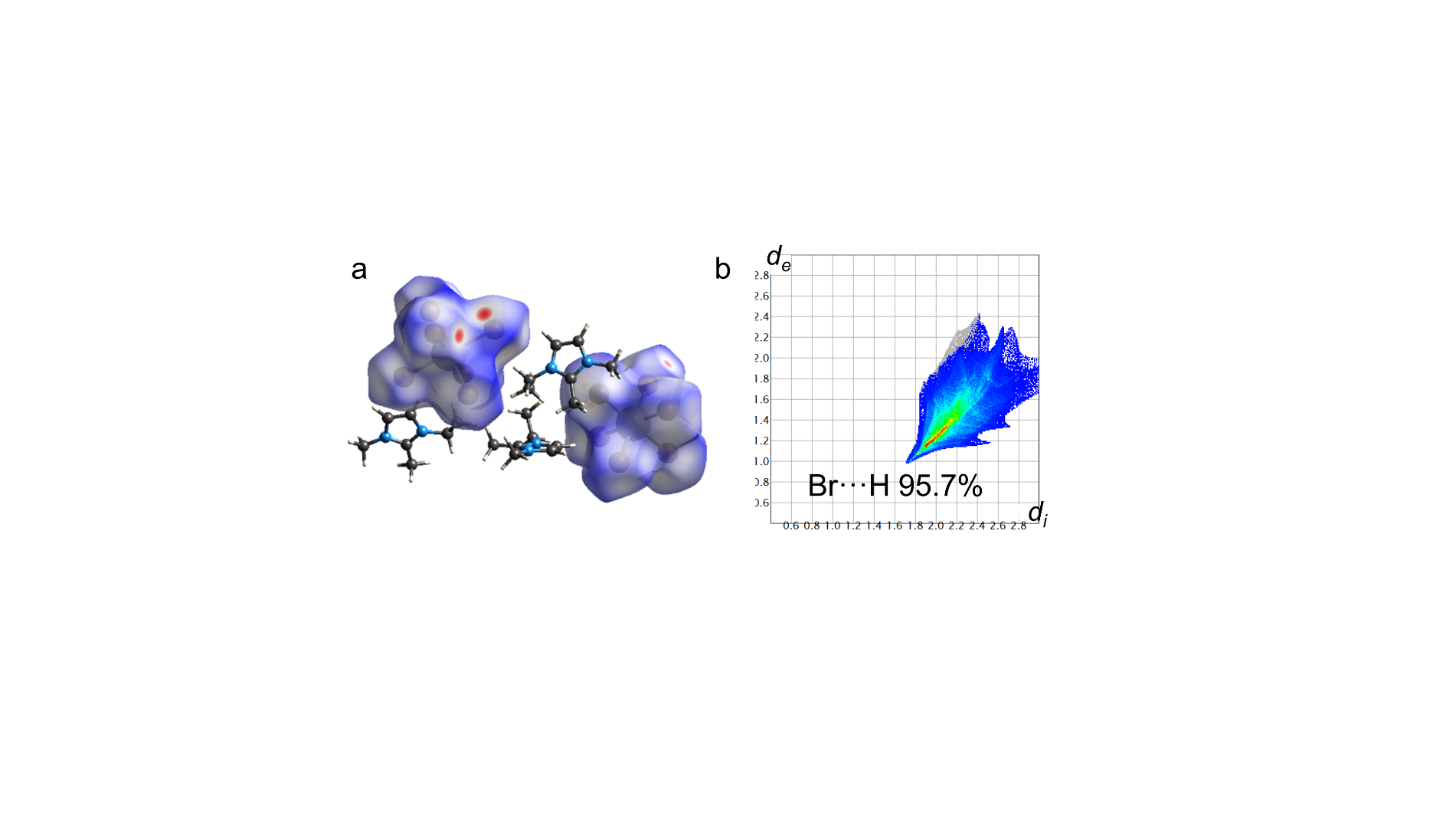


**Figure S4.** Hirshfeld surface analysis (a) and 2D fingerprint plot (b) of [Emmim]_3_CeBr_6_ showing Br···H interaction (95.7%).





**Figure S5.** X-ray refinement with difference Rietveld plot of [Emmim]_3_CeBr_6_.


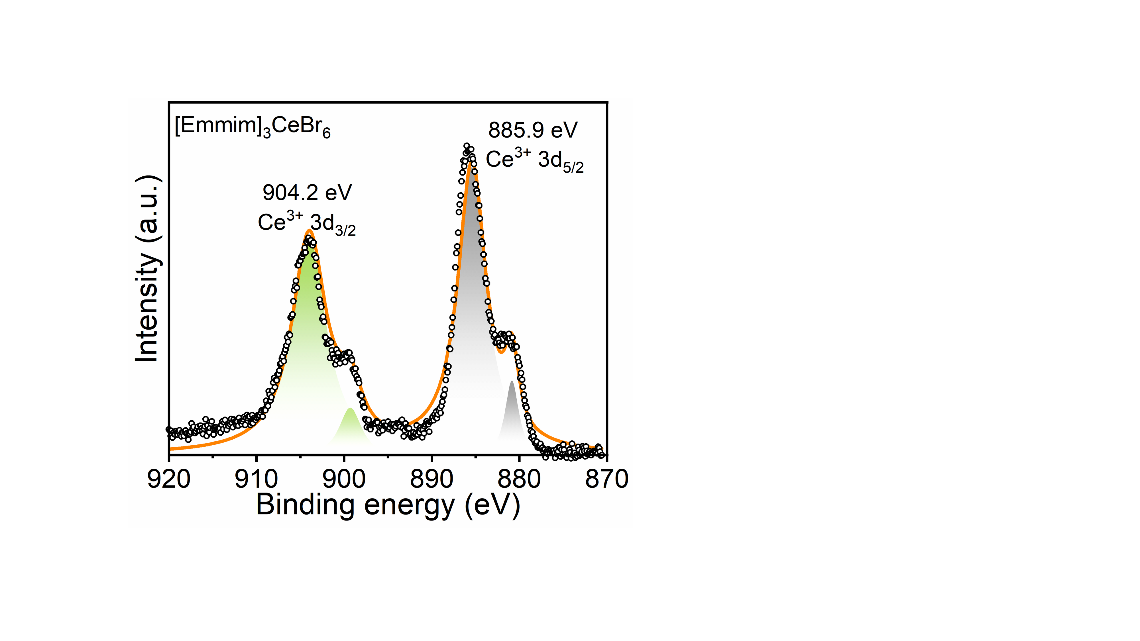


**Figure S6.** High-resolution XPS spectrum of Ce 3d for [Emmim]_3_CeBr_6_.


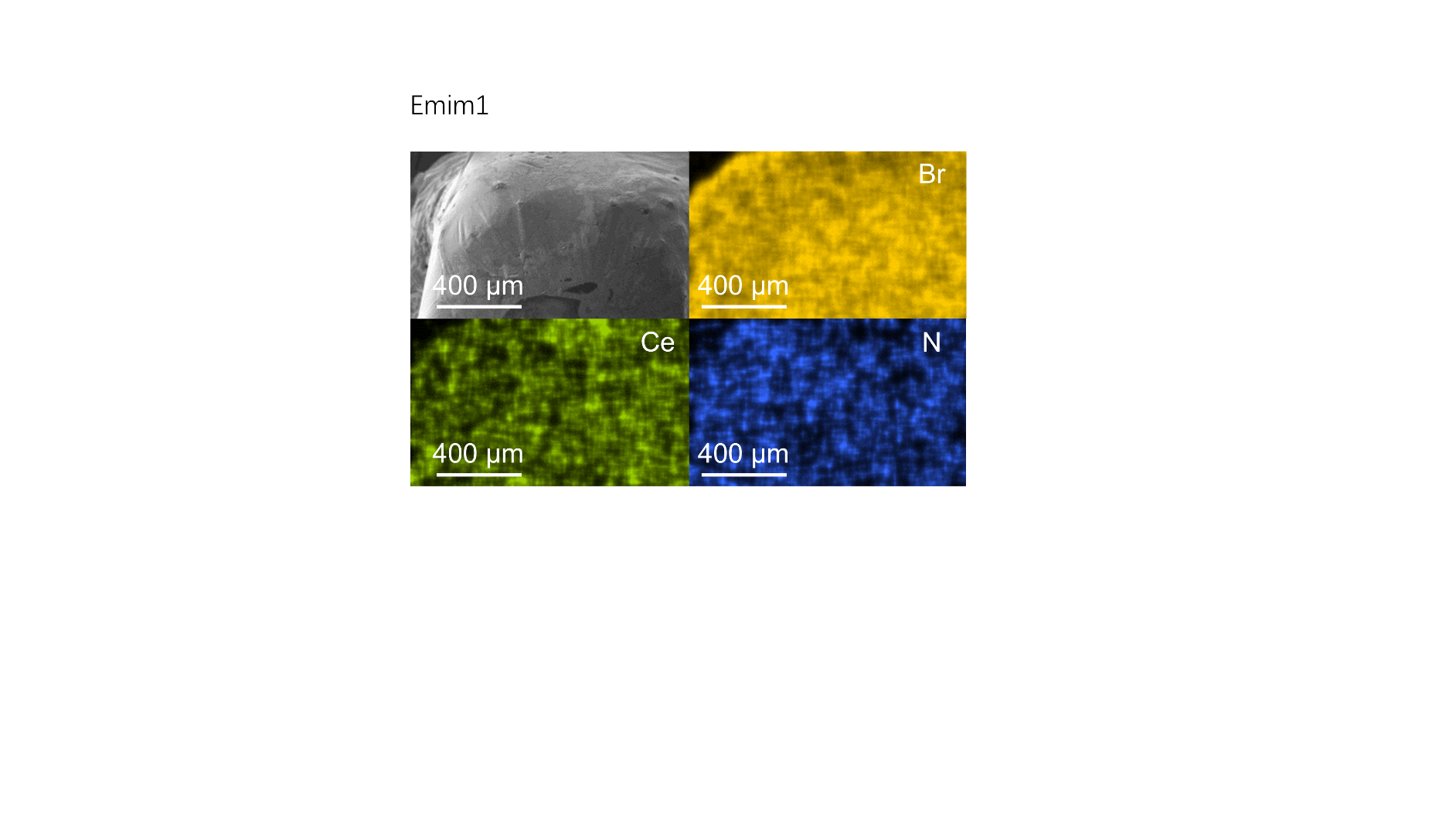


**Figure S7.** EDS elemental mapping images of N, Br, Ce and for [Emim]_3_CeBr_6_.


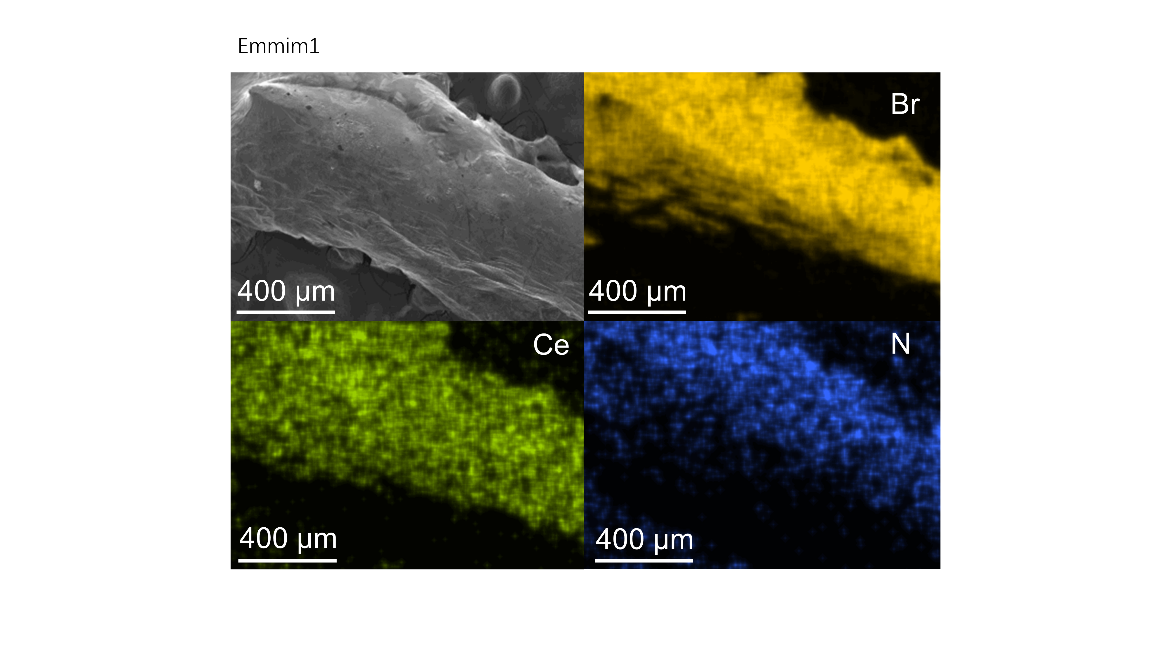


**Figure S8.** EDS elemental mapping images of N, Br, Ce and for [Emmim]_3_CeBr_6_.


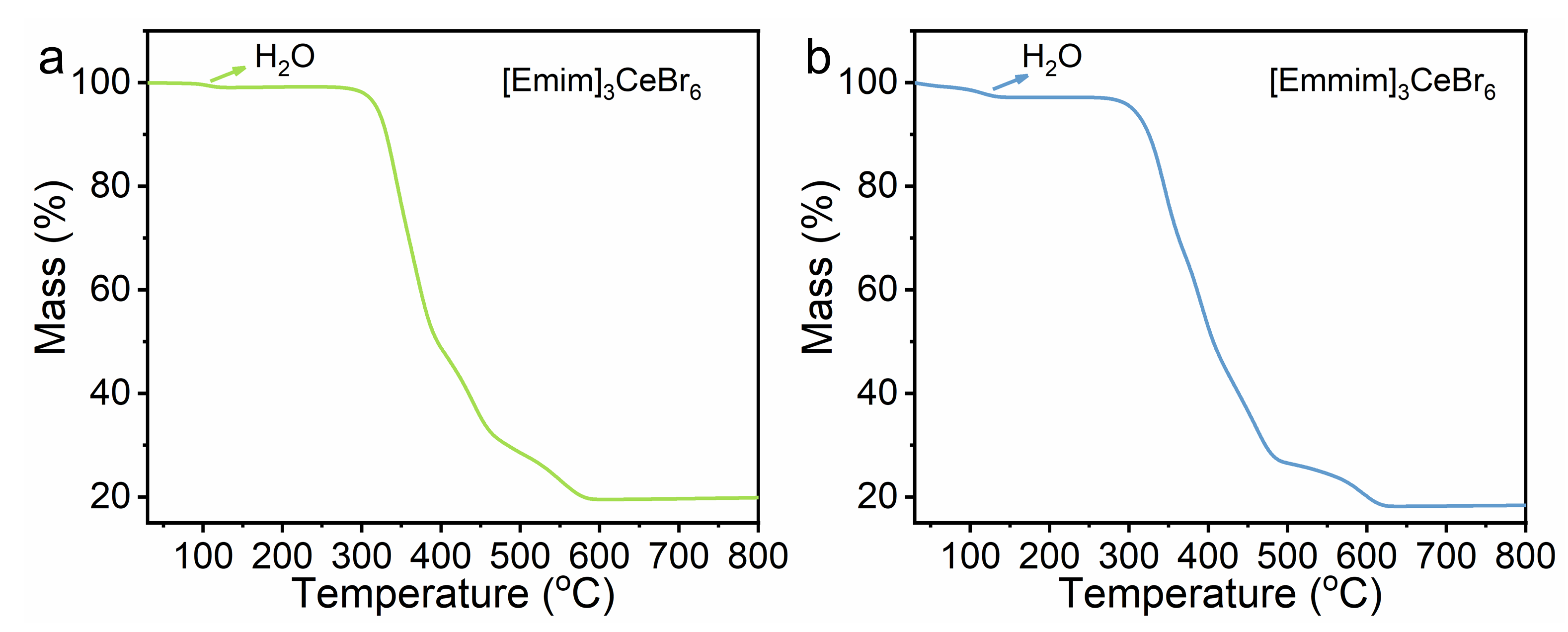


**Figure S9.** TG curves for [Emim]_3_CeBr_6_ (a) and [Emmim]_3_CeBr_6_ (b).

**
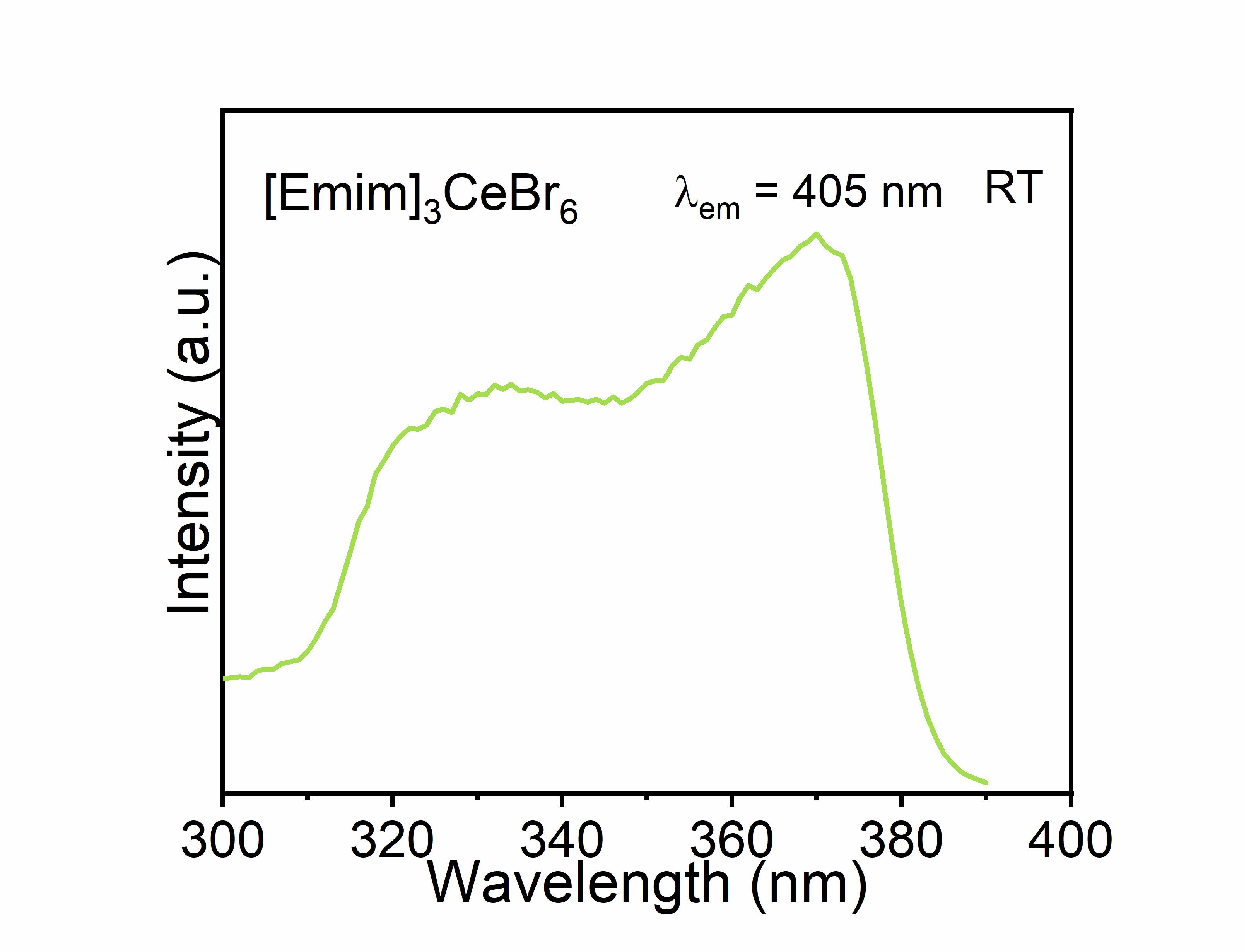
**

**Figure S10.** PLE spectrum at RT monitored at 405 nm emission for [Emim]_3_CeBr_6_.





**Figure S11.** PLE spectrum at RT monitored at 408 nm emission for [Emmim]_3_CeBr_6_.


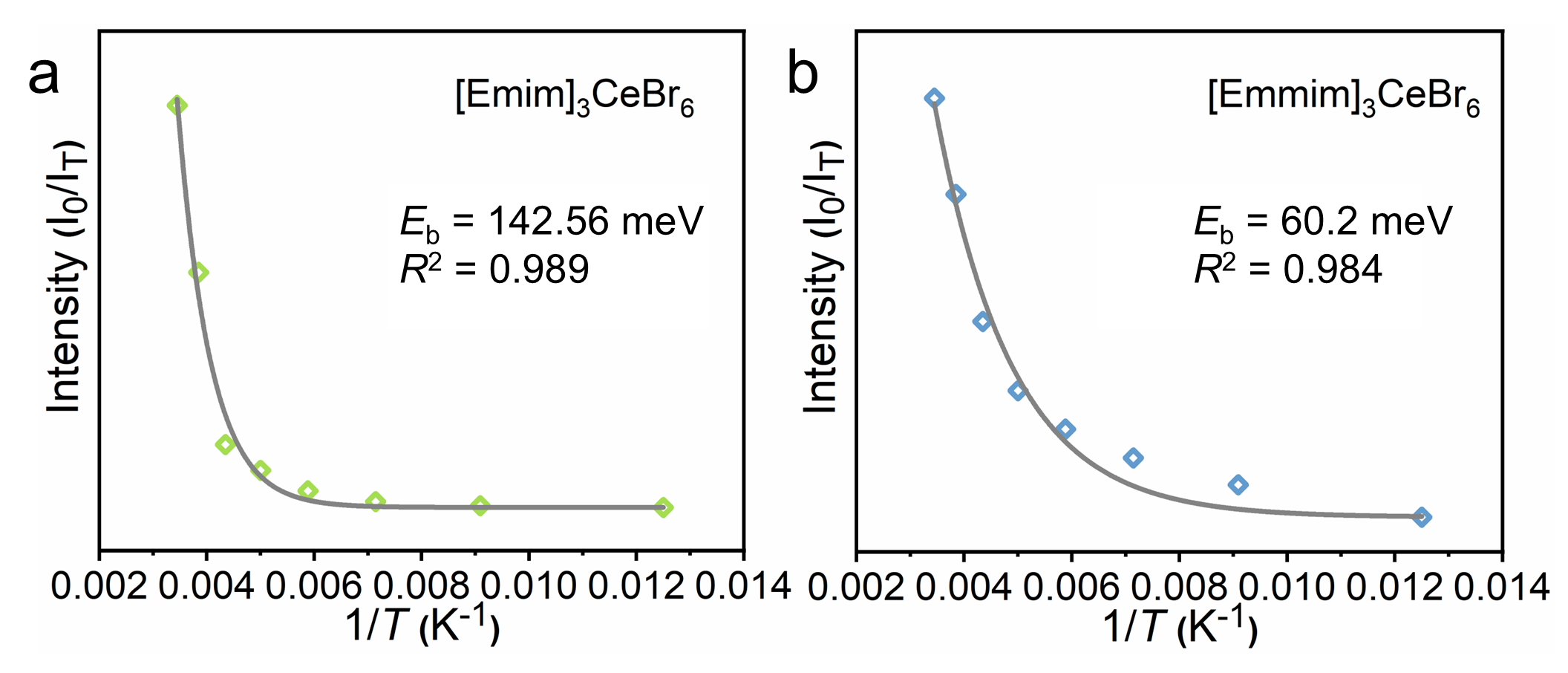


**Figure S12.** Fitted of the integrated intensity with temperature for [Emim]_3_CeBr_6_ (a) and [Emmim]_3_CeBr_6_ (b).





**Figure S13.** PL decay spectra at 80 K and RT upon 340 nm laser excitation and monitored at 408 nm emission for [Emim]_3_CeBr_6_.





**Figure S14.** PL decay spectra at 80 K and RT upon 340 nm laser excitation and monitored at 408 nm emission for [Emmim]_3_CeBr_6_.





**Figure S15.** Optical diffuse reflection spectrum of [Emim]_3_CeBr_6_.





**Figure S16.** Optical diffuse reflection spectrum of [Emmim]_3_CeBr_6_.


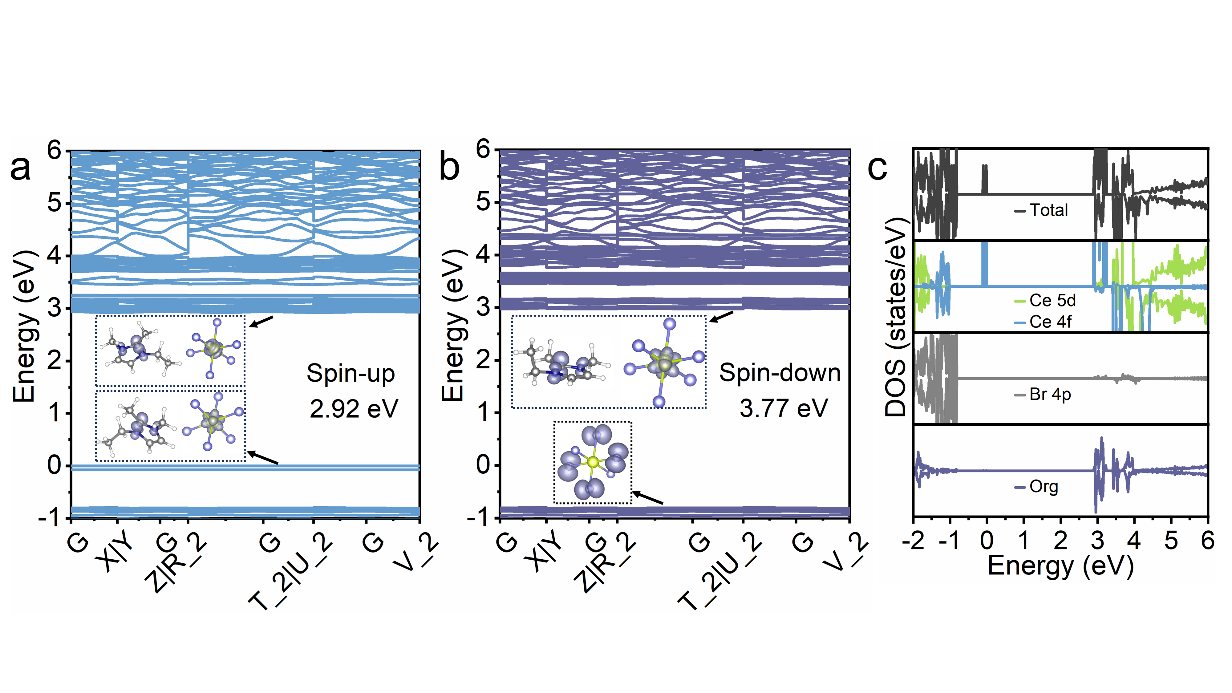


**Figure S17.** Calculated spin-up (a) and spin-down (b) band structure of [Emmim]_3_CeBr_6_ with the bandgap value of 2.92 and 3.77 eV, respectively; inset shows the charge-density at VBM and CBM. (c) Density of state (DOS) of [Emmim]_3_CeBr_6_.


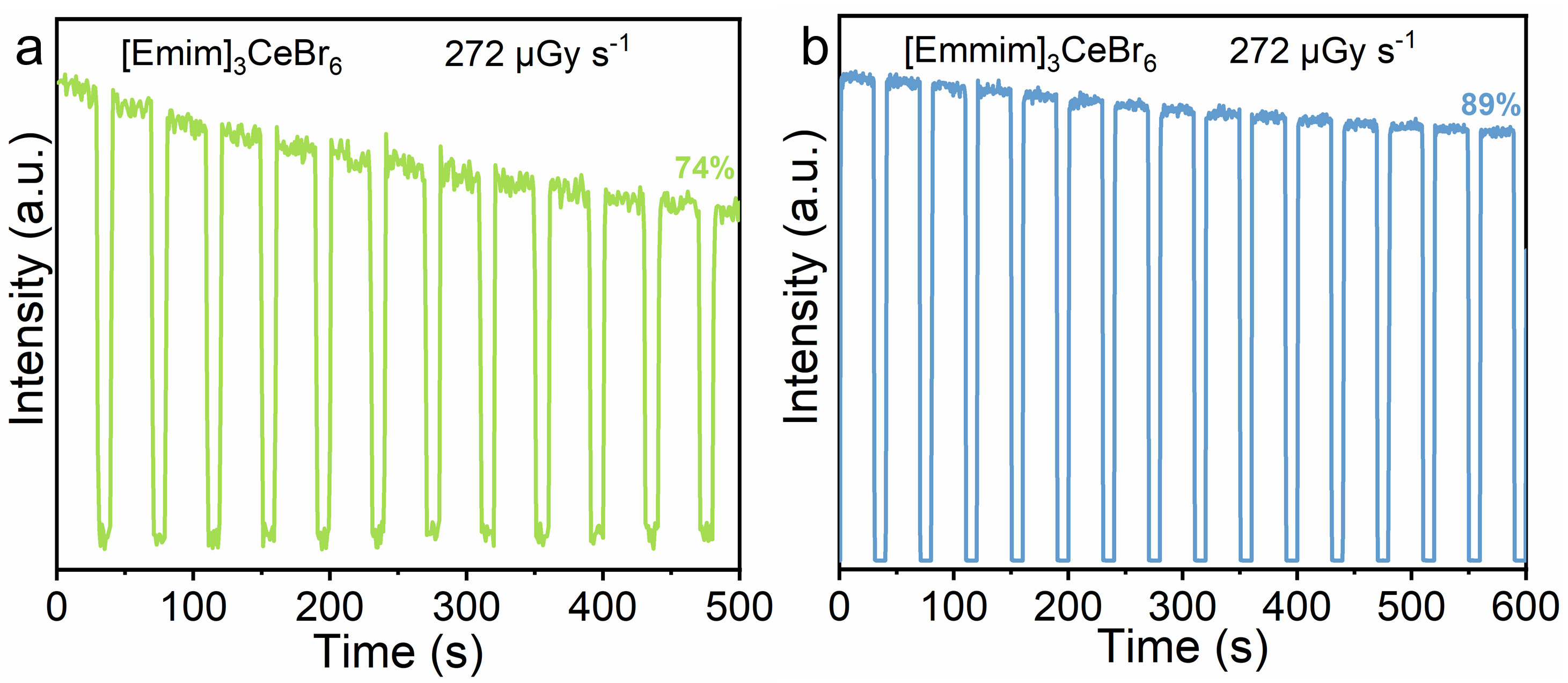


**Figure S18.** Radioluminescence of X-ray irradiation stability for [Emim]_3_CeBr_6_ (a) and [Emmim]_3_CeBr_6_ (b).


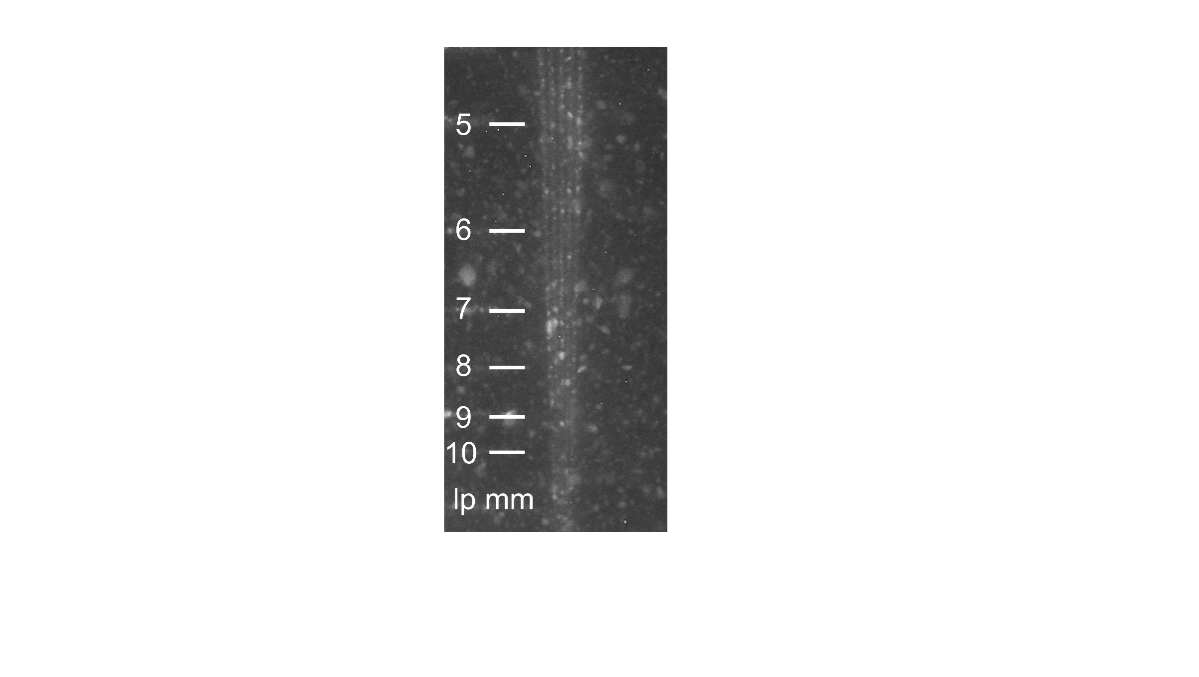


**Figure S19.** X-ray imaging of a standard line-pair card (lp mm^-1^) based on [Emmim]_3_CeBr_6_ thin film.

**Table S1**. Crystallographic data and refinement details for [Emim]_3_CeBr_6_ and [Emmim]_3_CeBr_6_.

| Compound | [Emim]_3_CeBr_6_ | [Emmim]_3_CeBr_6_ |
| --- | --- | --- |
| Empirical formula | CeBr_6_C_18_H_33_N_6_ | CeBr_6_C_21_H_39_N_6_ |
| Formula Mass | 953.08 | 995.16 |
| Crystal system | Monoclinic | Triclinic |
| Space group | *P*2_1_/*c* | *P*-1 |
| *a*/Å | 15.8919(5) | 10.04200(10) |
| *b*/Å | 12.7719(4) | 10.8313(2) |
| *c*/Å | 15.1052(5) | 15.8543(2) |
| *α*/° | 90 | 90.7380(10) |
| *β*/° | 90.458(3) | 92.1860(10) |
| *γ*/° | 90 | 106.9450(10) |
| *V*/Å^3^ | 3065.80(17) | 1647.85(4) |
| *Z* | 4 | 2 |
| *T*/K | 100(2) | 110(2) |
| *λ*/Å | 0.71073 | 0.71073 |
| *F*(000) | 1804 | 950 |
| *ρ*_calcd_/g cm^-3^ | 2.065 | 2.006 |
| *μ*/mm^-1^ | 9.322 | 8.676 |
| Measured refls. | 16334 | 30713 |
| Independent refls. | 5339 | 5795 |
| No. of parameters | 289 | 319 |
| *R*_int_ | 0.0214 | 0.0695 |
| *R*_1_ (*I*> 2*σ*(*I*))^a^ | 0.0357 | 0.0270 |
| *wR*(*F*^2^) (*I*> 2*σ*(*I*))^b^ | 0.0842 | 0.0642 |
| GOF | 1.048 | 1.057 |
| CCDC number | 2373675 | 2373676 |

**Table S2.** Selected bond lengths and angles for [Emim]_3_CeBr_6_.

| Bond /Angle | Value (Å) / (°) |
| --- | --- |
| Ce(1)-Br(1) | 2.9136(5) |
| Ce(1)-Br(1)#1 | 2.9136(5) |
| Ce(1)-Br(2) | 2.9162(5) |
| Ce(1)-Br(2)#1 | 2.9162(5) |
| Ce(1)-Br(3)#1 | 2.9174(6) |
| Ce(1)-Br(3) | 2.9174(6) |
| Ce(2)-Br(4)#2 | 2.8971(6) |
| Ce(2)-Br(4) | 2.8971(6) |
| Ce(2)-Br(6)#2 | 2.9093(5) |
| Ce(2)-Br(6) | 2.9094(5) |
| Ce(2)-Br(5) | 2.9246(5) |
| Ce(2)-Br(5)#2 | 2.9246(5) |
| Br(1)-Ce(1)-Br(1)#1 | 180.0 |
| Br(1)-Ce(1)-Br(2) | 89.384(15) |
| Br(1)#1-Ce(1)-Br(2) | 90.616(15) |
| Br(1)-Ce(1)-Br(2)#1 | 90.616(15) |
| Br(1)#1-Ce(1)-Br(2)#1 | 89.385(15) |
| Br(2)-Ce(1)-Br(2)#1 | 180.0 |
| Br(1)-Ce(1)-Br(3)#1 | 89.158(16) |
| Br(1)#1-Ce(1)-Br(3)#1 | 90.842(16) |
| Br(2)-Ce(1)-Br(3)#1 | 90.869(16) |
| Br(2)#1-Ce(1)-Br(3)#1 | 89.131(16) |
| Br(1)-Ce(1)-Br(3) | 90.842(16) |
| Br(1)#1-Ce(1)-Br(3) | 89.158(16) |
| Br(2)-Ce(1)-Br(3) | 89.131(16) |
| Br(2)#1-Ce(1)-Br(3) | 90.869(16) |
| Br(3)#1-Ce(1)-Br(3) | 180.0 |
| Br(4)#2-Ce(2)-Br(4) | 180.00(2) |
| Br(4)#2-Ce(2)-Br(6)#2 | 89.705(16) |
| Br(4)-Ce(2)-Br(6)#2 | 90.295(16) |
| Br(4)#2-Ce(2)-Br(6) | 90.294(16) |
| Br(4)-Ce(2)-Br(6) | 89.706(16) |
| Br(6)#2-Ce(2)-Br(6) | 180.0 |
| Br(4)#2-Ce(2)-Br(5) | 93.434(16) |
| Br(4)-Ce(2)-Br(5) | 86.566(16) |
| Br(6)#2-Ce(2)-Br(5) | 90.150(14) |
| Br(6)-Ce(2)-Br(5) | 89.851(14) |
| Br(4)#2-Ce(2)-Br(5)#2 | 86.566(16) |
| Br(4)-Ce(2)-Br(5)#2 | 93.434(16) |
| Br(6)#2-Ce(2)-Br(5)#2 | 89.850(14) |
| Br(6)-Ce(2)-Br(5)#2 | 90.149(14) |
| Br(5)-Ce(2)-Br(5)#2 | 180.00(3) |
| #1 -*x*+2, -*y*, -*z*+1; #2 -*x*+1, -*y*, -*z* | |

**Table S3.** Selected bond lengths and angles for [Emmim]_3_CeBr_6_.

| Bond /Angle | Value (Å) / (°) |
| --- | --- |
| Ce(1)-Br(1)#1 | 2.9049(4) |
| Ce(1)-Br(1) | 2.9049(4) |
| Ce(1)-Br(3)#1 | 2.9371(4) |
| Ce(1)-Br(3) | 2.9371(4) |
| Ce(1)-Br(2) | 2.9439(4) |
| Ce(1)-Br(2)#1 | 2.9439(4) |
| Ce(2)-Br(6) | 2.9227(4) |
| Ce(2)-Br(6)#2 | 2.9227(4) |
| Ce(2)-Br(4)#2 | 2.9241(4) |
| Ce(2)-Br(4) | 2.9241(4) |
| Ce(2)-Br(5) | 2.9356(4) |
| Ce(2)-Br(5)#2 | 2.9356(4) |
| Br(1)#1-Ce(1)-Br(1) | 180.0 |
| Br(1)#1-Ce(1)-Br(3)#1 | 87.100(11) |
| Br(1)-Ce(1)-Br(3)#1 | 92.900(11) |
| Br(1)#1-Ce(1)-Br(3) | 92.900(11) |
| Br(1)-Ce(1)-Br(3) | 87.100(11) |
| Br(3)#1-Ce(1)-Br(3) | 180.0 |
| Br(1)#1-Ce(1)-Br(2) | 92.969(10) |
| Br(1)-Ce(1)-Br(2) | 87.031(10) |
| Br(3)#1-Ce(1)-Br(2) | 87.412(10) |
| Br(3)-Ce(1)-Br(2) | 92.588(10) |
| Br(1)#1-Ce(1)-Br(2)#1 | 87.031(10) |
| Br(1)-Ce(1)-Br(2)#1 | 92.969(10) |
| Br(3)#1-Ce(1)-Br(2)#1 | 92.588(10) |
| Br(3)-Ce(1)-Br(2)#1 | 87.412(10) |
| Br(2)-Ce(1)-Br(2)#1 | 180.0 |
| Br(6)-Ce(2)-Br(6)#2 | 180.0 |
| Br(6)-Ce(2)-Br(4)#2 | 89.940(11) |
| Br(6)#2-Ce(2)-Br(4)#2 | 90.060(11) |
| Br(6)-Ce(2)-Br(4) | 90.059(11) |
| Br(6)#2-Ce(2)-Br(4) | 89.940(11) |
| Br(4)#2-Ce(2)-Br(4) | 179.999(17) |
| Br(6)-Ce(2)-Br(5) | 89.426(11) |
| Br(6)#2-Ce(2)-Br(5) | 90.575(11) |
| Br(4)#2-Ce(2)-Br(5) | 90.782(11) |
| Br(4)-Ce(2)-Br(5) | 89.218(11) |
| Br(6)-Ce(2)-Br(5)#2 | 90.574(11) |
| Br( 6)#2-Ce(2)-Br(5)#2 | 89.425(11) |
| Br(4)#2-Ce(2)-Br(5)#2 | 89.218(12) |
| Br(4)-Ce(2)-Br(5)#2 | 90.782(11) |
| Br(5)-Ce(2)-Br(5)#2 | 180.000(15) |
| #1 -*x*+1, -*y*+1, -*z*+1; #2 -*x*, -*y*+2, -*z*+2 | |

**Table S4.** Selected H-bond data for [Emim]_3_CeBr_6_.

| D−H···A | D−H (Å) | H···A (Å) | D···A (Å) | <(DHA) (°) |
| --- | --- | --- | --- | --- |
| C(1)-H(1)...Br(1) | 0.95 | 2.83 | 3.576(5) | 135.6 |
| C(2)-H(2)...Br(5)#3 | 0.95 | 2.91 | 3.742(7) | 146.4 |
| C(3)-H(3)...Br(4)#2 | 0.95 | 2.88 | 3.663(5) | 140.5 |
| C(4)-H(4B)...Br(3)#1 | 0.99 | 2.97 | 3.955(6) | 172.6 |
| C(5)-H(5A)...Br(4)#2 | 0.98 | 2.96 | 3.792(7) | 143.6 |
| C(6)-H(6A)...Br(3)#4 | 0.98 | 2.93 | 3.752(6) | 141.7 |
| C(6)-H(6B)...Br(1) | 0.98 | 3.08 | 3.897(6) | 142.0 |
| C(7)-H(7)...Br(5)#2 | 0.95 | 2.78 | 3.660(5) | 153.8 |
| C(8)-H(8)...Br(3)#4 | 0.95 | 2.84 | 3.717(6) | 153.6 |
| C(9)-H(9)...Br(6)#3 | 0.95 | 2.90 | 3.702(6) | 143.0 |
| C(10)-H(10A)...Br(6)#3 | 0.99 | 3.00 | 3.743(5) | 132.3 |
| C(10)-H(10B)...Br(6) | 0.99 | 3.04 | 3.785(5) | 133.4 |
| C(11)-H(11B)...Br(5)#5 | 0.98 | 3.11 | 4.001(5) | 151.8 |
| C(12)-H(12C)...Br(2)#6 | 0.98 | 2.96 | 3.677(6) | 131.0 |
| C(13)-H(13)...Br(1)#6 | 0.95 | 2.96 | 3.691(8) | 134.6 |
| C(14)-H(14)...Br(5)#5 | 0.95 | 2.86 | 3.680(7) | 145.6 |
| C(15)-H(15)...Br(2)#7 | 0.95 | 3.12 | 3.821(7) | 132.4 |
| C(16)-H(16A)...Br(2)#7 | 0.99 | 3.11 | 3.897(7) | 137.6 |
| C(17)-H(17C)...Br(1)#7 | 0.98 | 3.08 | 3.875(6) | 139.4 |
| C(18)-H(18B)...Br(2)#6 | 0.98 | 2.74 | 3.706(7) | 169.4 |
| C(18)-H(18C)...Br(4)#8 | 0.98 | 2.80 | 3.637(6) | 144.2 |
| Symmetry transformations used to generate equivalent atoms: #1 -*x*+2, -*y*, -*z*+1; #2 -*x*+1, -*y*, -*z*; #3 -*x*+1, *y*+1/2, -*z*+1/2; #4 *x*, -*y*+1/2, *z*-1/2; #5 *x*, *y*+1, *z*; #6 -*x*+2, -*y*+1/2, -*z*+1/2; #7 -*x*+2, -*y*+1, -*z*+1; #8 -*x*+1, -*y*+1, -*z*. | | | | |

**Table S5.** Selected H-bond data for [Emmim]_3_CeBr_6_.

| D−H···A | D−H (Å) | H···A (Å) | D···A (Å) | <(DHA) (°) |
| --- | --- | --- | --- | --- |
| C(2)-H(2)...Br(2)#3 | 0.95 | 3.09 | 3.752(4) | 128.6 |
| C(3)-H(3)...Br(1)#1 | 0.95 | 3.14 | 3.860(4) | 133.9 |
| C(3)-H(3)...Br(2) | 0.95 | 2.80 | 3.539(4) | 134.9 |
| C(4)-H(4A)...Br(4)#4 | 0.98 | 3.11 | 3.778(5) | 127.1 |
| C(5)-H(5A)...Br(2)#3 | 0.98 | 3.09 | 3.982(5) | 151.3 |
| C(6)-H(6A)...Br(4)#4 | 0.99 | 2.97 | 3.739(4) | 135.7 |
| C(6)-H(6A)...Br(6)#5 | 0.99 | 3.04 | 3.828(4) | 137.0 |
| C(6)-H(6B)...Br(1)#1 | 0.99 | 3.09 | 3.758(4) | 125.7 |
| C(7)-H(7A)...Br(1)#1 | 0.98 | 3.11 | 3.727(5) | 122.6 |
| C(10)-H(10)...Br(5)#2 | 0.95 | 3.10 | 3.919(4) | 145.8 |
| C(10)-H(10)...Br(6) | 0.95 | 3.11 | 3.783(4) | 129.1 |
| C(11)-H(11A)...Br(5)#4 | 0.98 | 3.12 | 3.687(4) | 118.4 |
| C(12)-H(12B)...Br(1)#6 | 0.98 | 2.85 | 3.805(5) | 165.6 |
| C(13)-H(13A)...Br(5)#2 | 0.99 | 3.04 | 3.957(5) | 154.0 |
| C(13)-H(13B)...Br(6)#5 | 0.99 | 2.93 | 3.914(4) | 173.9 |
| C(16)-H(16)...Br(2)#7 | 0.95 | 3.05 | 3.682(4) | 125.0 |
| C(16)-H(16)...Br(3)#8 | 0.95 | 3.00 | 3.849(4) | 149.0 |
| C(17)-H(17)...Br(2)#1 | 0.95 | 3.12 | 3.913(4) | 141.6 |
| C(18)-H(18A)...Br(4) | 0.98 | 2.94 | 3.660(4) | 130.8 |
| C(18)-H(18B)...Br(3)#9 | 0.98 | 3.11 | 3.608(4) | 112.9 |
| C(19)-H(19B)...Br(3)#8 | 0.98 | 3.13 | 4.058(5) | 158.2 |
| C(19)-H(19C)...Br(4) | 0.98 | 3.11 | 3.816(4) | 130.0 |
| C(20)-H(20A)...Br(2)#1 | 0.99 | 2.96 | 3.764(4) | 138.9 |
| C(21)-H(21A)...Br(3)#1 | 0.98 | 3.13 | 3.831(4) | 129.8 |
| Symmetry transformations used to generate equivalent atoms: #1 -*x*+1, -*y*+1, -*z*+1; #2 -*x*, -*y*+2, -*z*+2; #3 -*x*+1, -*y*, -*z*; #4 *x*, *y*-1, *z*; #5 -*x*, -*y*+1, -*z*+2; #6 -*x*, -*y*+1, -*z*+1; #7 *x*, *y*+1, *z*; #8 -*x*+1, -*y*+2, -*z*+1; #9 *x*-1, *y*, *z*. | | | | |

**Table S6.** Main parameters of processing and refinement of [Emim]_3_CeBr_6_ and [Emmim]_3_CeBr_6_.

| Sample | Space Group | Cell parameters (Å),  Cell Volume (Å^3^) | *R*_wp_, *R*_p_, *R*_B_, *χ*^2^ |
| --- | --- | --- | --- |
| [Emim]_3_CeBr_6_ | *P*2_1_/*c* | *a* = 15.9297(15),  *b* = 12.8594(12),  *c* = 15.5277(15),  *β* = 90.3947(60),  *V* = 3180.72(53) | 4.54, 3.64, 2.25, 1.57 |
| [Emmim]_3_CeBr_6_ | *P*-1 | *a* = 10.12262(96),  *b* = 10.9073(11),  *c* = 16.0247(14),  *α* = 90.9241(69),  *β* = 92.2434(48),  *γ* = 106.7314(60),  *V* = 1692.39(28) | 4.3, 3.23, 1.98, 1.53 |

**Table S7.** Light yield and lifetime for Mn^2+^, Cu^+^, and Eu^2+^ based X-ray metal halide scintillators.

| Compound | Light yield (ph MeV^-1^) | Lifetime (μs) | Reference |
| --- | --- | --- | --- |
| Mn^2+^ based halides | | |  |
| [C_4_H_12_N]MnCl_3_ | 50500 | 758.95 | [1] |
| [C_8_H_20_N]_2_MnBr_4_ | 24400 | 442.52 |  |
| [PrPP]_2_MnBr_4_ | 43511 | 355 | [2] |
| [TPP]_2_MnBr_4_ ceramic | 78000 | 265 | [3] |
| [ETP]_2_MnBr_4_ | 35000 | 295 | [4] |
| Cu^+^ based halides | | |  |
| [MTP]_2_Cu_4_I_6_ glass ceramic | 64000 | 1.9 | [5] |
| [C_8_H_20_N]_2_Cu_2_Br_4_ ceramic | 91300 | 56 | [6] |
| [TBA]CuCl_2_ | 23373 | 28.7 | [7] |
| [TBA]CuBr_2_ | 24134 | 232.05 |  |
| Rb_2_CuCl_3_ | 16600 | 11.3 | [8] |
| Rb_2_CuBr_3_ | 91056 | 41.4 | [9] |
| Eu^2+^ based halides | | |  |
| [PP14]EuBr_3_MeOH | 24000 | 0.370 | [10] |
| [PP13]EuBr_3_MeOH | 19000 | 0.312 |  |
| [Emim]EuBr_3_MeOH | 43000 | 0.382 |  |
| [BA]_10_EuI_12_ | 27000 | 0.108 | [11] |

**Table S8.** Light yield and lifetime for Ce^3+^ based gamma-ray metal halide scintillators.

| Compound | Light yield (ph MeV^-1^) | Lifetime (μs) | Reference |
| --- | --- | --- | --- |
| CeF_3_ | 4500 | 0.028 | [12] |
| LuF_3_:Ce^3+^ | 8000 | 0.028 | [13] |
| LuI_3_:Ce^3+^ | 99000 | 0.033 | [14] |
| LaCl_3_:Ce^3+^ | 49000 | 0.025 | [15] |
| LaBr_3_:Ce^3+^ | 67000 | 0.015 | [16] |
| CeCl_3_ | 28000 | 0.025 | [17] |
| K_2_LaCl_5_:Ce^3+^ | 30000 | 1 | [18] |
| K_2_LaBr_5_:Ce^3+^ | 40000 | 0.100 |  |
| RbGd_2_Cl_7_:Ce^3+^ | 40000 | 0.090 | [19] |
| RbGd_2_Br_7_:Ce^3+^ | 56000 | 0.043 | [20] |
| GdBr_3_:Ce^3+^ | 44000 | 0.020 | [21] |
| GdI_3_:Ce^3+^ | 47000 | 0.045 | [22] |

## References

[1] T. Jiang, W. Ma, H. Zhang, Y. Tian, G. Lin, W. Xiao, X. Yu, J. Qiu, X. Xu, Y. Yang, D. Ju, Highly Efficient and Tunable Emission of Lead-Free Manganese Halides toward White Light-Emitting Diode and X-Ray Scintillation Applications Adv. Funct. Mater., (2021) 2009973.

[2] S.B. Xiao, X. Zhang, X. Mao, H.J. Yang, Z.N. Chen, L.J. Xu, Ultrahigh X-Ray Imaging Spatial Resolution Enabled by an 0D Mn(II) Hybrid Scintillator Adv. Funct. Mater., (2024) 2404003.

[3] K. Han, K. Sakhatskyi, J. Jin, Q. Zhang, M.V. Kovalenko, Z. Xia, Seed-Crystal-Induced Cold Sintering Toward Metal Halide Transparent Ceramic Scintillators Adv. Mater., 34 (2022) 2110420.

[4] B. Li, Y. Xu, X. Zhang, K. Han, J. Jin, Z. Xia, Zero-Dimensional Luminescent Metal Halide Hybrids Enabling Bulk Transparent Medium as Large-Area X-Ray Scintillators Adv. Opt. Mater., 10 (2022) 2102793.

[5] B. Li, J. Jin, X. Liu, M. Yin, X. Zhang, Z. Xia, Y. Xu, Multiphase Transformation in Hybrid Copper(I)-Based Halides Enable Improved X-ray Scintillation and Real-Time Imaging ACS Materials Lett., 6 (2024) 1542-1548.

[6] B. Su, J. Jin, K. Han, Z. Xia, Ceramic Wafer Scintillation Screen by Utilizing Near-Unity Blue-Emitting Lead-Free Metal Halide (C_8_H_20_N)_2_Cu_2_Br_4_ Adv. Funct. Mater., (2022) 2210735.

[7] L. Lian, X. Wang, P. Zhang, J. Zhu, X. Zhang, J. Gao, S. Wang, G. Liang, D. Zhang, L. Gao, H. Song, R. Chen, X. Lan, W. Liang, G. Niu, J. Tang, J. Zhang, Highly Luminescent Zero-Dimensional Organic Copper Halides for X-ray Scintillation J. Phys. Chem. Lett., 12 (2021) 6919-6926.

[8] X. Zhao, G. Niu, J. Zhu, B. Yang, J.-H. Yuan, S. Li, W. Gao, Q. Hu, L. Yin, K.-H. Xue, E. Lifshitz, X. Miao, J. Tang, All-Inorganic Copper Halide as a Stable and Self-Absorption-Free X-ray Scintillator J. Phys. Chem. Lett., 11 (2020) 1873-1880.

[9] B. Yang, L. Yin, G. Niu, J.-H. Yuan, K.-H. Xue, Z. Tan, X.-S. Miao, M. Niu, X. Du, H. Song, E. Lifshitz, J. Tang, Lead-Free Halide Rb_2_CuBr_3_ as Sensitive X-Ray Scintillator Adv. Mater., 31 (2019) 1904711.

[10] J. Jin, K. Han, Y. Wang, Z. Xia, Bandgap Narrowing in Europium(II)-Based Bromide Hybrids toward Improved X-ray Scintillation and Imaging Chem. Mater., 36 (2024) 4813-4820.

[11] X. Zhao, P. Fu, P. Li, H. Du, J. Zhu, C. Ge, L. Yang, B. Song, H. Wu, T. Jin, Q. Guo, L. Wang, J. Li, Z. Xiao, J. Chang, G. Niu, J. Luo, J. Tang, Solution-Processed Hybrid Europium (II) Iodide Scintillator for Sensitive X-Ray Detection Research, 6 (2023) 0125.

[12] W.W. Moses, S.E. Derenzo, Cerium Fluoride, a New Fast, Heavy Scintillator IEEE T Nucl. Sci., 36 (1989) 173-176.

[13] B. Moine, C. Dujardin, H. Lautesse, C. Pedrini, C.M. Combes, A. Belsky, P. Martin, J.Y. Gesland, Spectroscopic and Scintillation Properties of Cerium-Doped LuF_3_ Single Crystal Mater. Sci. Forum, 245 (1997) 239-241.

[14] M.D. Birowosuto, P. Dorenbos, C.W.E. van Eijk, K.W. Krämer, H.U. Güdel, High-light-output scintillator for photodiode readout: LuI_3_:Ce^3+^ J. Appl. Phys., 99 (2006) 123520.

[15] E.V.D. van Loef, P. Dorenbos, C.W.E. van Eijk, K. Krämer, H.U. Güdel, High-energy-resolution scintillator: Ce^3+^ activated LaCl_3_ Appl. Phys. Lett., 77 (2000) 1467-1468.

[16] E.V.D. van Loef, P. Dorenbos, C.W.E. van Eijk, K. Krämer, H.U. Güdel, High-energy-resolution scintillator: Ce^3+^ activated LaBr_3_ Appl. Phys. Lett., 79 (2001) 1573-1575.

[17] S.E. Derenzo, W.W. Moses, Heavy Scintillators for Scientific and Industrial Application Proc. Crystal 2000 International Workshop (Edition Frontiéres, Gif-sur-Yvette, France), (1993) 125.

[18] E.V.D. van Loef, P. Dorenbos, C.W.E. van Eijk, K.W. Krämer, H.U. Güdel, Scintillation properties of K_2_LaX_5_:Ce^3+^ (X=Cl, Br, I) Nucl. Instrum. Meth. A, 537 (2005) 232-236.

[19] M.D. Birowosuto, P. Dorenbos, Novel γ- and X-ray scintillator research: on the emission wavelength, light yield and time response of Ce^3+^ doped halide scintillators Phys. Status Solidi A, 206 (2009) 9-20.

[20] P. Dorenbos, J.C. van’t Spijker, O.W.V. Frijns, C.W.E. van Eijk, K. Krgmer, H.U. Giidel, A. Ellens, Scintillation properties of RbGd_2_Br_7_: Ce^3+^ crystals; fast, efficient, and high density scintillators Nucl. Instrum. Meth. B, 132 (1997) 728-731.

[21] E.V.D. van Loef, P. Dorenbos, C.W.E. van Eijk, K.W. Krämer, H.U. Güdel, Optical and scintillation properties of pure and Ce^3+^ doped GdBr_3_ Opt. Commun., 189 (2001) 297-304.

[22] M.D. Birowosuto, P. Dorenbos, G. Bizarri, C.W.E. van Eijk, K.W. Kramer, H.U. Gudel, Temperature Dependent Scintillation and Luminescence Characteristics of GdI_3_: Ce^3+^ IEEE T Nucl. Sci., 55 (2008) 1164-1169.
